# Determination of Anti-Alzheimer’s Disease Activity of Selected Plant Ingredients

**DOI:** 10.3390/molecules27103222

**Published:** 2022-05-18

**Authors:** Tomasz Tuzimski, Anna Petruczynik

**Affiliations:** 1 Department of Physical Chemistry, Medical University of Lublin, Chodźki 4a, 20-093 Lublin, Poland; 2 Department of Inorganic Chemistry, Medical University of Lublin, Chodźki 4a, 20-093 Lublin, Poland

**Keywords:** anti-Alzheimer’s disease activity, plant extract components, enzyme activity inhibition, IC_50_ values

## Abstract

Neurodegenerative diseases, among which one of the more common is Alzheimer’s disease, are the one of the biggest global public health challenges facing our generation because of the increasing elderly population in most countries. With the growing burden of these diseases, it is essential to discover and develop new treatment options capable of preventing and treating them. Neurodegenerative diseases, among which one of the most common is Alzheimer’s disease, are a multifactorial disease and therefore demand multiple therapeutic approaches. One of the most important therapeutic strategies is controlling the level of acetylcholine—a neurotransmitter in cholinergic synapses—by blocking the degradation of acetylcholine using acetylcholinesterase inhibitors such as tacrine, galantamine, donepezil and rivastigmine. However, these drugs can cause some adverse side effects, such as hepatotoxicity and gastrointestinal disorder. Thus, the search for new, more effective drugs is very important. In the last few years, different active constituents from plants have been tested as potential drugs in neurodegenerative disease therapy. The availability, lower price and less toxic effects of herbal medicines compared with synthetic agents make them a simple and excellent choice in the treatment of neurodegenerative diseases. The empirical approach to discovering new drugs from the systematic screening of plant extracts or plant-derived compounds is still an important strategy when it comes to finding new biologically active substances. The aim of this review is to identify new, safe and effective compounds that are potential candidates for further in vivo and clinical tests from which more effective drugs for the treatment of Alzheimer’s disease could be selected. We reviewed the methods used to determine anti-Alzheimer’s disease activity. Here, we have discussed the relevance of plant-derived compounds with in vitro activity. Various plants and phytochemical compounds have shown different activity that could be beneficial in the treatment of Alzheimer’s disorders. Most often, medicinal plants and their active components have been investigated as acetylcholinesterase and/or butyrylcholinesterase activity inhibitors, modifiers of β-amyloid processing and antioxidant agents. This study also aims to highlight species with assessed efficacy, usable plant parts and the most active plant components in order to identify species and compounds of interest for further study. Future research directions are suggested and recommendations made to expand the use of medicinal plants, their formulations and plant-derived active compounds to prevent, mitigate and treat Alzheimer’s disease.

## 1. Introduction

Neurodegenerative diseases are a heterogeneous group of disorders that are characterized by the progressive degeneration of the structure and function of the central nervous system or the peripheral nervous system. Common neurodegenerative diseases include Alzheimer’s disease and Parkinson’s disease. Alzheimer’s disease is a chronic progressive neurodegenerative disorder that leads to the selective deterioration of cholinergic neurons in the basal forebrain [1]. Patients with Alzheimer’s disease are characterized as having difficulties with cognition, such as loss of memory and reasoning disabilities due to a decrease in neuronal activity and decreased concentrations of neurotransmitters in intersynaptic space, causing poor synaptic transmission, which leads to a deficit in cholinergic neurotransmission in the central nervous system. 

The pathogenicity of Alzheimer’s disease is complex and includes genetic and environmental factors and therefore demands multiple therapeutic approaches. Some of the more well-known processes involved in Alzheimer’s disease pathogenesis include cholinergic deficit, oxidative stress, inflammatory pathways (especially NFκB) and the hyperphosphorylation and aggregation of tau proteins and β and γ secretases responsible for APP processing [2]. The most important changes observed in the brain are a decrease in cortical levels of the neurotransmitter acetylcholine. In Alzheimer’s disease patients, acetylcholine has a very short half-life due to the presence of large amounts of acetylcholinesterase, an enzyme involved in the metabolic hydrolysis of acetylcholine at cholinergic synapses in the central and peripheral nervous system [3]. Alzheimer’s disease, which is the most common form of neurodegenerative disorder, is also the result of the accumulation of amyloid-β peptide into microscopic “plaques” and the twisting of tau proteins into strands of dead and dying neurons. The early stages of Alzheimer’s disease are also associated with inflammation and oxidative stress [4].

**Figure 1:**
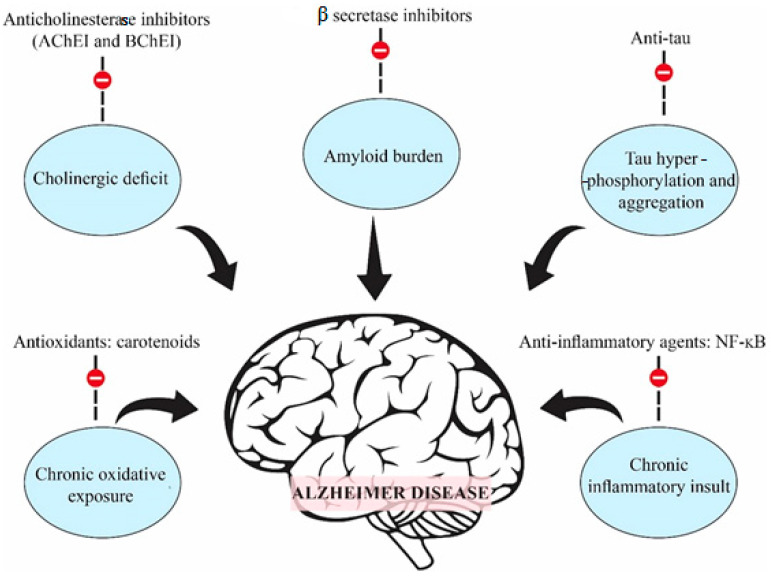
Schematic diagram showing five of the most important therapeutic targets in Alzheimer’s disease. Reprinted with permission from Ref. [2]. Copyright 2021 Elsevier.

The treatment of Alzheimer’s disease is limited to symptomatic and palliative medications and, despite the enormous effort aimed at understanding the etiology and pathogenesis of the disease, no drugs have so far been able to effectively stop for a long time the progression of the disease. Figure 1 presents schematic diagram showing five of the most important therapeutic targets in Alzheimer’s disease [2]. The treatment of Alzheimer’s disease has as its main aim an increase in the levels of acetylcholine in the synaptic cleft by inhibiting cholinesterase enzymes, which are responsible for the degradation of acetylcholine. The low levels of acetylcholine in patients with Alzheimer’s disease are associated with symptoms to low memory, memory loss and gradual learning decrease [5]. Until now, no drug of choice for the treatment of this disease has been decided. 

Currently, only a few drugs, such as galantamine, donepezil, tacrine and rivastigmine, have been approved to treat Alzheimer’s disease. These drugs temporarily improve symptoms associated with Alzheimer’s disease through the upregulation of neurotransmitter chemicals located in the brain [6]. The main disadvantage of these drugs is their moderate and temporal benefit, lasting for a maximum of 12–24 months. Additionally, the approved drugs do not reduce the rate of decline in cognitive or functional capacities in the long term. Furthermore, adverse reactions to currently used drugs are often more serious than the disease itself. 

Plants are rich resources of different bioactive constituents that can be used for the treatment of several diseases. Especially, traditional medicinal plants have served as a repository of bioactive constituents, and they are the basis for new drug search. Therefore, the empirical approach to discover new drugs from the systematic screening of plant extracts or plant-derived substances remains an important strategy for finding new biologically active compounds. Currently, there are a large number of natural compounds of plant origin, such as alkaloids, terpenoids and phenolic compounds, with neuroprotective effects, which are considered for use in the treatment of neurodegenerative diseases, including Alzheimer’s disease or Parkinson’s disease. 

Nowadays, in vitro and in vivo methods are used for screening compounds that can be potentially used for the treatment of neurodegenerative diseases. However, in vivo screening methods are time-consuming, waste a significant amount of human and material resources and usually consume large amounts of raw material [7]. The in vitro method is rapid and usually requires only a small amount of raw material, and it is suitable for the preliminary selection of active components from a variety of medicinal herbs and foods. 

Cytotoxicity testing is also very important for safety assessment extracts from medicinal plants and for the search for new active compounds. Cytotoxicity testing is important when it comes to assessing and validating the safety of medicinal plants for traditional use, and it serves as a guide in the quest for novel active compounds. Investigations into the cytotoxicity of extracts and active compounds are evaluated in normal cell lines. For further investigation, extracts and their active components that exhibit the highest possible activity against Alzheimer’s disease and, at the same time, show the lowest toxicity should be selected.

In recent years, some review articles on the screening of compounds with potential therapeutic approaches in treating Alzheimer’s disease have been published. Most of them discuss single methods for a selected, often narrow group of plant compounds or selected plant families. The application of flavonoids as acetylcholinesterase inhibitors was reviewed by Khan et al. [5]. The authors focused on the relevance of plant-derived flavonoids with preclinical activity, mechanisms of action and structural activity relationship, which might to lead to novel effective acetylcholinesterase inhibitors. The usefulness of African medicinal plants in improving cognition and memory in Alzheimer’s disease patients was also described [8]. The authors stated that several commonly used African medicinal plants inhibited acetylcholinesterase activity, modified β-amyloid processing, protected oxidative stress and regulated antioxidant enzyme activity. The review provides a compilation of medicinal plants that could be further studied for their bioactive constituents, which may become safe, effective and novel therapeutic candidates for the treatment of neurodegenerative diseases. The neuropharmacology effects, including, among others, acetylcholinesterase antioxidant, anti-inflammatory inhibition and β-amyloid reduction properties, of Nigella sativa and its main component, thymoquinone, were reviewed [9]. Natural β-carboline alkaloids as a privileged scaffold for multitarget strategies in Alzheimer’s disease therapy were also reviewed [10]. Acetylcholinesterase activity inhibition, butyrylcholinesterase activity inhibition, β-amyloid aggregation inhibition, monoamine oxidases inhibition, 5-hydroxytryptamine receptor binding and other activity of the natural β-carboline derivatives were described [10].

This review provides a compilation of medicinal plants that could be further investigated for their bioactive constituents; these may become novel therapeutic candidates for the treatment of neurodegenerative diseases. Our search results showed medicinal plants and their possible mechanisms of action including the inhibition of acetylcholinesterase and butyrylcholinesterase activities, the modification of β-amyloid processing, anti-inflammatory activity and protection against oxidative stress. 

In the first part of the review, the most important therapeutic targets in Alzheimer’s disease, such as the inhibition of cholinesterase activity, the inhibition of amyloid fibrils production, the inhibition of monoamine oxidases, pancreatic lipase, tyrosinase, inflammatory effects and antioxidant activity, will be discussed. 

### 1.1. Inhibition of Acetylcholinesterase Activity 

Enzymes play several important roles in the homeostasis of living organisms, catalyzing important physiological reactions. In the control of diseases, it is possible to use the strategy of inhibiting the activity of a certain enzyme to improve the clinical condition of a specific disease. Cholinesterases include two types of enzymes, namely acetylcholinesterase and butyrylcholinesterase. Acetylcholinesterase preferentially hydrolyzes acetylcholine, while butyrylcholinesterase hydrolyzes butyrylcholine more efficiently than acetylcholine. Additionally, acetylcholinesterase is mostly of neuronal origin, while butyrylcholinesterase is primarily present in the blood and glial cells [11]. 

Acetylcholinesterase is a serine hydrolase enzyme whose main function is to modulate cholinergic signal transmission through the hydrolysis of acetylcholine. The enzyme catalyzes the hydrolysis of the neurotransmitter acetylcholine into the two inactive compounds choline and acetic acid [12,13]. 

**Figure 2:**
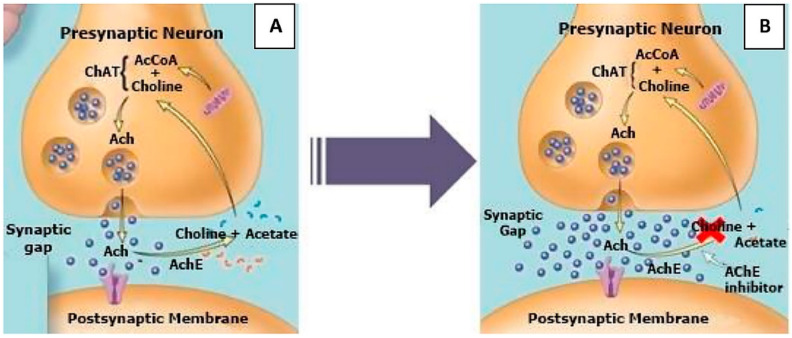
General scheme of the cholinergic hypothesis for AChE inhibition. (**A**) Low concentrations of acetylcholine in the synaptic gap. (**B**) Increase in concentration after inhibition of AChE. Reprinted with permission from Ref. [13]. Copyright 2020 Elsevier.

The scheme of the cholinergic hypothesis for acetylcholinesterase inhibition is presented in Figure 2 [13]. Acetylcholinesterase is primarily responsible for the termination of the nerve impulse transmission at the cholinergic synapses through catalyzing the hydrolysis of the neurotransmitter acetylcholine and accelerates the aggregation of β-amyloid peptides. An important strategy for treating Alzheimer’s disease is to keep the levels of acetylcholine in the synaptic cleft by blocking the acetylcholinesterase. The inhibition of acetylcholinesterase in the brain leads to an increase in acetylcholine concentration and partly restores the substantial impairment of memory and cognitive dysfunctions. Furthermore, some research has shown that acetylcholinesterase induces amyloid fibril formation by interaction throughout the peripherical anionic site of the enzyme, forming highly toxic acetylcholinesterase–β-amyloid peptide complexes [14].

Most of the drugs currently used for treating Alzheimer’s disease (galanthamine, donezepil, tacrine, rivastigmine) are inhibitors of acetylcholinesterase activity [2]. 

One of the most important therapeutic strategies in the treatment of neurodegenerative diseases is controlling the level of acetylcholine, as a neurotransmitter in cholinergic synapses, through blocking the degradation of acetylcholine using acetylcholinesterase inhibitors [15]. Therefore, important issues for finding new efficient treatments of neurodegenerative diseases may be the discovery of effective acetylcholinesterase activity inhibitors.

Acetylcholinesterase inhibition activity is traditionally tested by spectrophotometry method described by Ellman [16]. It is a method based on the reaction of Ellman’s reagent (5,5-dithiobis-(2-nitrobenzoic acid)) with a thiol group of substrates formed by the action of acetylcholinesterase. The reaction of Ellman’s reagent with thiol groups leads to the formation of 2-nitro-5-mercaptobenzoic acid, which is further hydrolyzed in the water at neutral or alkaline pH. Free 2-nitro-5-mercaptobenzoic acid has a yellow color. Currently, measurements are most often carried out with the application of 96-well microplate [17,18], rarely using a spectrophotometer [19]. 

Often, for the evaluation of acetylcholinesterase inhibition activity, besides the spectrophotometric method, in silico investigations using quantitative structure–activity relationship (QSAR) model analysis and molecular docking are performed [13]. QSARs are important determinants of safety and efficacy after the primary screening of compounds, help in lead optimization and are therefore critical for the drug discovery and development process [2].

Currently, chromatography, especially thin-layer chromatography (TLC), is increasingly applied for determination of acetylcholinesterase activity inhibition. The advantages of the TLC method are that there is no disturbance from sample-dissolving solvents, as in the microplate assay, and it is a very simple method. TLC also allows to the simple elimination of the mobile phase after development. Additionally, the minimal detectable amount for an acetylcholinesterase inhibitor tested was much less than that needed for the microplate assay. TLC is compatible with various chemical (derivatization reagents), biochemical (enzymatic) and biological (cell-based) assays that can be performed directly in situ in the adsorbent layer in the method applied for the determination of acetylcholinesterase activity inhibition, where samples are loaded onto TLC plates. TLC has been successfully applied for the determination of acetylcholinesterase inhibition activity [20,21]. In the procedure, the plates were sprayed with acetylcholinesterase solution, dried, and incubated usually at 37 °C. The enzymatic activity was detected by spraying with a solution of 1-naphthyl acetate in ethanol and aqueous solution of fast blue B salt according to method proposed by Marston et al. [22]. Potential acetylcholinesterase inhibitors appeared as bright zones on a purple background. Chromatography combined with assays can be utilized to link to individual components of a complex matrix such as a plant extract without a time-consuming and expensive stepwise isolation procedure. The spectrophotometric method using microplate readers allows analyzing many samples quickly at the same time, but deeper colors of samples would increase the values of absorbance, resulting in absorbance values exceeding 0.8 easily, and cannot be measured accurately, while the method for the determination of acetylcholinesterase activity inhibition by HPLC has been proved to be simple and feasible and eliminates the problem caused by deep color. HPLC combines the advantages of high sensitivity, selectivity and wide application with the elimination of the color interference caused by changes in pH. High-performance liquid chromatography (HPLC) with on-line coupled UV, mass spectrometric and biochemical detection was applied for the identification of acetylcholinesterase inhibitors from natural products [23]. Wang et al. also used HPLC for testing the acetylcholinesterase inhibitory activity of anthocyanins. These compounds present markedly diverse colors in different values of pH, which makes it difficult to determine the absorbance in the color-rendering experiment [24]. Online acetylcholinesterase activity inhibition determination by high-performance liquid chromatography–mass spectrometry (LC-MS) hyphenated with an immobilized enzyme reactor has also been rarely applied [25]. Ultrafiltration liquid chromatography-mass spectrometry (UFLC–MS) is an also efficient method that can be applied to rapidly screen and identify ligands binding to acetylcholinesterase [7].

Nature provides a large number of bioactive compounds with high acetylcholinesterase inhibitory activity. The need arises for the development of new acetylcholinesterase inhibitors with lower toxicity and more potent activity. The majority of studies have focused on acetylcholinesterase inhibition by alkaloids belonging to various classes. The other major classes of compound reported to have anti-acetylcholinesterase activity are the components of essential oils such as terpenoids and various phenolic compounds. 

### 1.2. Inhibition of Butyrylcholinesterase Activity 

Butyrylcholinesterase is mainly involved in the breakdowns of butyrylcholine. The enzyme acetylcholinesterase predominates in the healthy brain, with butyrylcholinesterase considered to play a minor role in regulating brain acetylcholine levels. For this reason, under normal conditions, acetylcholine is dominantly hydrolyzed by acetylcholinesterase. However, when the level of acetylcholinesterase in cholinergic transmission declines, butyrylcholinesterase can play a function compensation role for acetylcholinesterase to some extent to maintain normal cholinergic pathways [11]. Butyrylcholinesterase activity progressively increases in patients with Alzheimer’s disease, while acetylcholinesterase activity remains unchanged or declines [26]. Both enzymes, therefore, represent legitimate therapeutic targets for ameliorating the cholinergic deficit considered to be responsible for the declines in cognitive, behavioral and global functioning characteristic of Alzheimer’s disease. The dual inhibition of acetylcholinesterase and butyrylcholinesterase is beneficial for Alzheimer’s disease patients, especially since butyrylcholinesterase replaces acetylcholinesterase in the acetyl choline catabolism in advanced Alzheimer’s disease patients [26].

Butyrylcholinesterase inhibitors also increase choline levels for the reduction in Alzheimer’s disease symptoms. In the later stages of Alzheimer’s disease, acetylcholinesterase activity is downregulated by up to 33–45% of normal values, while the activity of butyrylcholinesterase is improved by 40–90% in certain brain regions [27]. However, except for rivastigmine, which is a dual acetylcholinesterase-butyrylcholinesterase inhibitor, approved selective acetylcholinesterase inhibitor drugs are not suitable for late-stage Alzheimer’s disease since acetylcholine hydrolysis in the late stage of the disease mainly depends on butyrylcholinesterase but not acetylcholinesterase [11]. Butyrylcholinesterase has a multitude of hydrolyzing activities, not only nonspecific cholinesterase activity but also acylamidase and peptidase activities [28]. The peptidase activity of butyrylcholinesterase is important because it is believed to be involved in the development and progression of Alzheimer’s disease, as it is a causative factor in the production of β-amyloids.

For this reason, an important issue in searching for new efficient treatments of neurodegenerative diseases may be the discovery of very effective butyrylcholinesterase inhibitors.

Most often, for the in vitro determination of butyrylcholinesterase activity inhibition by various plant extracts and plant-derived compounds, the modified Ellman method performed in 96-well plates is applied. Often, for this purpose, the Ellman method and molecular docking are simultaneously used.

Butyrylcholinesterase activity inhibition properties often show compounds isolated from plant extracts such as alkaloids and components of essential oils such as terpenoids and various phenolic compounds. 

The dual inhibition of acetylcholinesterase and butyrylcholinesterase by various plant extracts and their components may be considered a potential therapeutic advantage for neurodegenerative disease to benefit in cognition, global function, and behavioral symptoms, especially in advanced Alzheimer’s disease patients.

### 1.3. Inhibition of Amyloid Fibrils Production

Neurodegenerative diseases are also characterized by the extraneuronal accumulation of β-amyloid peptide. The accumulation of aggregated proteins at neurons has been correlated with Alzheimer’s disease patients, who show the presence of amyloid fibrils in the brain, which indicates the relationship between amyloid fibrils and the disease [19]. The accumulation of this toxic peptide leads to the deposition of β-amyloid into plaques and is thought to drive a pathologic cascade, which culminates in neuronal death [29]. These fibrils accumulate in the brain cells and central nervous system of Alzheimer’s disease patients and contribute to the symptoms of dementia. In histopathological investigations, the extracellular formation of senile plaques and intraneuronal appearance of neurofibrillary tangles, which are, respectively, due to the accumulation of β-amyloid peptides and hyperphosphorylated tau, are observed [30]. The amyloidogenic pathway is the consequence of the cleavage of β-amyloid precursor protein by the two β-amyloid-forming β- and γ-secretases that release 39–43-amino acid-long β-amyloid peptides, which are key players in the progression of Alzheimer’s disease. The presence of large α-amylase units in postmortem entorhinal cortex from Alzheimer’s disease patients and nondemented controls were also determined. The inhibition of amyloid fibrils production by plant extracts and compounds isolated from plants have been tested much less frequently than the acetylcholinesterase and butyrylcholinesterase activity inhibition.

β secretase initiates the production of the toxic β-amyloid that plays a crucial role in Alzheimer’s disease pathogenesis. β secretase, widely known as β-site amyloid precursor protein cleaving enzyme 1, cleaves the amyloid precursor protein in the first step in β-amyloid peptide production. The inhibition of β secretase is a prime therapeutic target for lowering cerebral β-amyloid concentrations in Alzheimer’s disease, and currently, the clinical development of β secretase inhibitors is being pursued. 

Various plant extracts containing different active compounds belonging to, e.g., phenolic compounds, saponins and triglycerides, have been tested as amyloid fibrils production inhibitors. The amyloid fibril production inhibitory properties have often been determined using electron microscopy, investigations on various cell lines and the determination of β secretase activity inhibition.

Monoamine oxidase catalyzes the oxidative deamination of biogenic amines and has an important role in the metabolism of neuroactive and vasoactive amines in the central nervous system and peripheral tissues. The enzyme preferentially degrades benzylamine and phenylethylamine and targets a wide variety of specific neurotransmitters involved in the primary substrates of monoamine oxidase in the brain, including epinephrine, norepinephrine, dopamine, serotonin and β-phenylethylamine. Activated monoamine oxidase also contributes to β-amyloid aggregation by two successive cleft β-secretase and γ-secretase of amyloid precursor protein. Additionally, activated monoamine oxidase is involved in the aggregation of neurofibrillary tangles and cognitive destruction through cholinergic neuronal damage and disorder in the cholinergic system. Monoamine oxidase inhibition has a general anti-Alzheimer’s disease effect as a consequence of oxidative stress reduction prompted by monoamine oxidase enzymes.

The effect of monoamine oxidase inhibition in vitro was tested using the method described by Green and Haughton often with slight modification [31]. In brief, the reaction mixture contained phosphate buffer at pH 7.4, semicarbazide, tyramine hydrochloride, enzyme and a solution of inhibitor. The mixture was usually incubated for 30 min. Next, acetic acid was added, and the mixture was usually incubated for 3 min in a boiling water bath followed by centrifugation. The resultant supernatant was mixed with equal volume of 2,4-dinitrophenylhydrazine, and benzene was added after incubation at room temperature. After separating the benzene layer, it was mixed with an equal volume of NaOH solution. The alkaline layer was decanted and incubated at 80 °C. The orange-yellow color developed was measured at 450 nm spectrophotometrically [31]. In in vitro investigations, PC12 cells from rat pheochromocytoma are often used in the study of neurodegenerative disease, as they have characteristics similar to midbrain dopamine neurons [7]. This cell line provides the advantages of rapid screening and short preparation time, and positive results can be verified during the primary neuronal culture. In PC12 cells, neuronal damage can be induced by β-amyloid (25–35), making PC12 cells effective in investigations of β-amyloid inhibition.

### 1.4. Inhibition of Monoamine Oxidases 

Monoamine oxidases catalyze the oxidative deamination of pharmacologically important monoamine neurotransmitters and are present as two monoamine oxidase isoforms (monoamine oxidase A and monoamine oxidase B) in the outer mitochondrial membranes of most tissues, including the brain. Monoamine oxidase (MAO) catalyzes the oxidative deamination of biogenic and xenobiotic amines and has an important role in the metabolism of neuroactive and vasoactive amines in the central nervous system (CNS) and peripheral tissues. Monoamine oxidase is critically related to amyloid plaque formation in Alzheimer’s disease patients, and monoamine oxidase B is expressed at high levels in the brain of patients with Alzheimer’s disease [32]. However, excessive monoamine oxidase inhibition, which can occur as a result of an excessive intake of enzyme inhibitors, is also harmful. Serotonin toxicity may cause a serious pathological disorder resulting from hyperactivity of serotonin neurotransmitter as a result of an excessive accumulation of serotonin due to excessive monoamine oxidase inhibition.

### 1.5. Inhibition of Pancreatic Lipase

Emerging evidence indicates that elevated cholesterol and triglyceride levels precede Alzheimer’s disease pathology [33]. Obesity and high levels of plasma cholesterol in middle age are related to a higher risk of Alzheimer’s disease, and elevated plasma triglyceride levels also precede amyloid deposition in Alzheimer’s disease mouse models.

### 1.6. Inhibition of Tyrosinase

Tyrosinase is a copper enzyme that plays an essential role in melanin biosynthesis in skin and hair and has also been proposed to contribute to the formation of neuromelanin [34]. The levels of the enzyme striatal-enriched protein tyrosine phosphatase are also raised in several different neurodegenerative disorders, including Alzheimer’s disease, fragile X syndrome and schizophrenia. The enzyme striatal-enriched protein tyrosine phosphatase normally opposes the development of synaptic strengthening, and these abnormally high levels of active enzyme striatal-enriched protein tyrosine phosphatase disrupt synaptic function by removing phosphate groups from a number of proteins, including several glutamate receptors and kinases. Dephosphorylation results in the internalization of glutamate receptors and inactivation of kinases—events that disrupt the consolidation of memories [35]. 

Most often, plant extracts containing phenolic compounds have been tested as tyrosinase inhibitors. For the in vitro determination of tyrosinase activity inhibition, various modifications of the spectrophotometric dopachrome method have been applied. 

### 1.7. Inhibition of Inflammatory Effects

Inflammation represents the defensive reaction of an organism to harmful exogenous or endogenous factors through the immune system. Inflammatory processes are involved in the onset and maintenance of many severe disorders including neurodegenerative diseases such as Alzheimer’s disease. Currently, increasing evidence highlights the important role of neuroinflammation in the degenerative process of neurodegenerative diseases. In Alzheimer’s disease, activated microglia and astrocytes are attracted and activated by β-amyloid plaques and release a series of pro-inflammatory mediators, resulting in inflammatory responses that may further damage neuronal cells, stimulate β-amyloid synthesis and increase microglial activation through a positive feedback loop [36]. Nitric oxide (NO) is a signaling molecule that plays a key role in the pathogenesis of inflammation. It gives an anti-inflammatory effect under normal physiological conditions. On the other hand, NO is considered a pro-inflammatory mediator that induces inflammation due to overproduction in abnormal situations. NO is synthesized and released into endothelial cells by the help of nitric oxide synthases that convert arginine into citrulline, producing NO in the process.

### 1.8. Antioxidant Activity

Reactive oxygen species and oxygen-centric free radicals such as hydroxyl radical, superoxide radical and hydrogen peroxide cause tissue damage and then cell death, which can oxidize lipids, proteins and DNA. Humans are unavoidably and continually affronted by different environmental stresses caused by reactive oxygen species, which induce pathological processes of many neurodegenerative diseases [37]. Nervous systems are understood to be principally susceptible to oxidative stress due to their limited antioxidant capacity, the utilization of metabolic oxygen, the failure of their neurons to synthesize glutathione and their high lipid content. 

Glutathione S-transferase was suggested as an important contributor to Alzheimer’s disease. As a group of the key antioxidant enzymes, glutathione S-transferases regulate the maintenance of glutathione and cellular detoxification and are involved in the activation of signals in cell apoptosis. 

Oxidative stress is defined as an imbalance between the concentration of oxidant (reactive oxygen species) and antioxidative defense mechanisms in favor of the oxidants. Oxidative stress can cause neuronal injury and death, and plays an important role in Alzheimer’s disease [38]. 

Various tests are applied for the determination of antioxidant activity. The 2,2-diphenyl-1-picrylhydrazyl (DPPH) free radical is a stable free radical that has been widely used as a tool for estimating the free radical scavenging activity of antioxidant. It measures the activity of an antioxidant to directly scavenge DPPH• by spectrophotometrically determining its absorbance at 515 nm [39].

In the ABTS assay, also known as Trolox equivalent antioxidant capacity assay, the green-blue stable radical cationic chromophore 2,2-azinobis-(3-ethylbenzothiazoline-6-sulfonate) (ABTS•^+^) is produced by oxidation and has absorption maxima at 414, 645, 734 and 815 nm. In the original assay, metmyoglobin was first reacted with H_2_O_2_ to generate the ferrylmyoglobin radical, which was then reacted with ABTS to form the ABTS•^+^. Subsequently, many modifications of the assay were introduced.

The ferric reducing antioxidant power assay is based on the reduction of a colorless Fe^3+^-2,4,6-Tris(2-pyridyl)-s-triazine complex into intense blue Fe^2+^-2,4,6-Tris(2-pyridyl)-s-triazine once it interacts with a potential antioxidant. At low cost, this method has shown to be useful for the screening of antioxidant capacities and comparing the efficiencies of different compound complexes into intense blue Fe^2+^-t2,4,6-Tris(2-pyridyl)-s-triazine once they interact with a potential antioxidant.

The β-carotene-linoleic acid model system method is based on the discoloration of β-carotene by the peroxides generated during the oxidation of linoleic acid at elevated temperature [39]. The β-carotene linoleic acid assay measures the inhibition of the volatile organic compounds and conjugated diene hydroperoxides arising from linoleic acid oxidation. 

Most often, antioxidant properties are determined for extracts containing phenolic compounds, and rarely for other compounds such as alkaloids and essential oil components. For the in vitro determination of the activity, often, the DPPH radical scavenging assay, ABTS radical scavenging assay, hydroxyl radical scavenging assay, ferric reducing antioxidant power assay, NO radical scavenging assay, metal chelating assay and cupric ion reducing assay are applied.

## 2. Determination of Anti-Neurodegenerative Disease Activity of Plant Compounds 

This section will discuss the groups of plant-derived chemicals showing high in vitro activity against Alzheimer’s disease, taking into account the methods used to determine this activity. The presence of active compounds in plant extracts and the activity of selected extracts will also be discussed.

### 2.1. Alkaloids

Currently, the most commonly used drugs for the treatment of neurodegenerative diseases contain alkaloids as an active ingredient, whose action lies in the inhibition of acetylcholinesterase. For this reason, it is promising to evaluate other alkaloids from various families of plants. A large number of alkaloids have been isolated from plants, of which some are tested for their possible acetylcholinesterase inhibition potential. Most often as potential anti-neurodegenerative disease drugs, alkaloids belonging to the steroidal, isoquinoline and indole classes, have been considered. Among these natural products, alkaloids are considered to be the most promising candidates for the treatment of neurodegenerative diseases due to their complex nitrogen-containing structures. Alkaloids, especially in high concentrations, can exhibit toxic properties, but investigations of alkaloids toxicity have rarely been conducted. A number of acetylcholinesterase and butyrylcholinesterase activity inhibitors have been isolated from various plant extracts, as extensively described in the literature.

Indole alkaloids, geissoschizoline, geissoschizone, geissospermine and 3′,4′,5′,6′-tetradehydrogeissospermine isolated from stembarks of Geissospermum vellosii, a native tree of Brazil, were tested as acetylcholinesterase inhibitors by Ellman’s method performed in a 96-well plate [36]. The highest anti-cholinesterase activity was observed for 3′,4′,5′,6′-tetradehydrogeissospermine with IC_50_ = 0.45 μM, but in cell viability tests (performed on mouse microglia N9 cell line), only geissoschizoline was not cytotoxic. For geissoschizoline in an experiment with Electrophorus electricus acetylcholinesterase, IC_50_ = 5.86 μM, and with human acetylcholinesterase, IC_50_ = 20.40 μM. Investigated indole alkaloids from Geissospermum vellosii were also evaluated as butyrylcholinesterase inhibitors [36]. IC_50_ values obtained by modified Ellman method in experiments with equine serum butyrylcholinesterase were from 0.32 μM obtained for 3′,4′,5′,6′-tetradehydrogeissospermine to 82.98 μM for geissospermine. In experiments with human butyrylcholinesterase, IC_50_ obtained for geissoschizoline was equal 10.21 μM. Both enzyme kinetic studies showed that geissoschizoline presented a mixed-type inhibition mechanism. The molecular docking simulation performed for acetylocholinesterase and butyrylcholinesterase shows that geissoschizoline interacts with both active site and peripheral anionic site, which suggest a dual site inhibitor profile. The anti-inflammatory activity of investigated alkaloids was also evaluated [36]. For the determination of anti-inflammatory activity, N9 cells cultured in 96-well plates were treated with lipopolysaccharide and geissoschizoline for 48 h. NO production was assessed by the determination of nitrite levels using the colorimetric Griess method [40]. An ELISA assay of inflammatory mediators was also performed. In the investigations, N9 cells cultured in 96-well plates were treated with lipopolysaccharide, physostigmine and geissoschizoline for 24 h. The levels of tumor necrosis factor alpha were determined in the cell supernatant, using enzyme immune-assay kits. Geissosquizoline showed an anti-inflammatory role, reducing the microglial release of NO and TNF-α at a concentration 20-fold lower than acetylcholinesterase IC_50_ and 10-fold lower than butyrylcholinesterase IC_50_. Indole alkaloid geissosquizoline, due to its multifunctional activity, can be useful in preventing neurodegeneration and restoring neurotransmission. 

Rocha et al. investigated other indole alkaloids isolated from Aspidosperma subincanum as acetylcholinesterase inhibitors [21]. An extract obtained from leaves and branches was investigated using the Elleman method in a 96-well microplate, with thin-layer chromatography and in silico prediction. The determination of inhibitory activity by thin-layer chromatography was performed on silica gel plates, which was eluted with a mixture of chloroform and methanol. The enzyme solution was sprayed, and the air-dried plate was incubated in a humid chamber at 37 °C for 20 min. Detection was performed by spraying the plate with a solution of 1-naphthyl acetate. The anticholinesterase activity was indicated by the presence of white spots on a purple background. The in vitro inhibition of acetylcholinesterase activity by extracts from the plant was moderate. The lowest IC_50_ values were obtained for dichloromethane extract from branches (77.88 µg/mL). The in silico prediction showed that the indole alkaloid uleine, its derivatives, olivacine derivatives 3,4-dihydroolivacine and N-methyl-tetrahydro-olivacine (guatambuine), and the subincanadines C and E demonstrated the possibility of inhibiting the activity of acetylcholinesterase. The investigated plant extracts were also tested for antioxidant activity [21]. The activity was determined using DPPH assay and β-carotene/linoleic acid co-oxidation assay. In the β-carotene bleaching assay, the ethanolic extract obtained from branches was able to prevent β-carotene bleaching with IC_50_ value of 39.0 μg/mL and was comparable to the IC_50_ value obtained in the experiment for rutin (39.4 μg/mL), but none of the tested extracts showed a significant capacity to reduce the DPPH radical (IC_50_ values > 15 μg/mL).

The extracts obtained from *Ocotea percoriacea* containing alkaloids isocorydine N-oxide, isocorydine N-oxide derivative, palmatine, roemerine and roemerine N-Oxide were in vitro tested for acetylcholinesterase activity inhibition. The determination of anti-cholinesterase activity was performed by modified Ellman method with the application of 96-well microplate [41]. Only hexane and dichlorometane fractions of Ocotea percoriacea extracts at concentration 1 mg/mL showed high inhibition activity after 30 min from the beginning of the reaction (83.28% and 92.09%, respectively). Ethanolic, buthanolic and aqueous fractions exhibited significantly lower activity. In silico studies performed for the alkaloids isolated from Ocotea percoriacea extracts suggest that the alkaloids bind as classical drugs such as tacrine and donepezil in the main binding sites and that they can be considered compounds with therapeutic potential.

**Figure 3:**
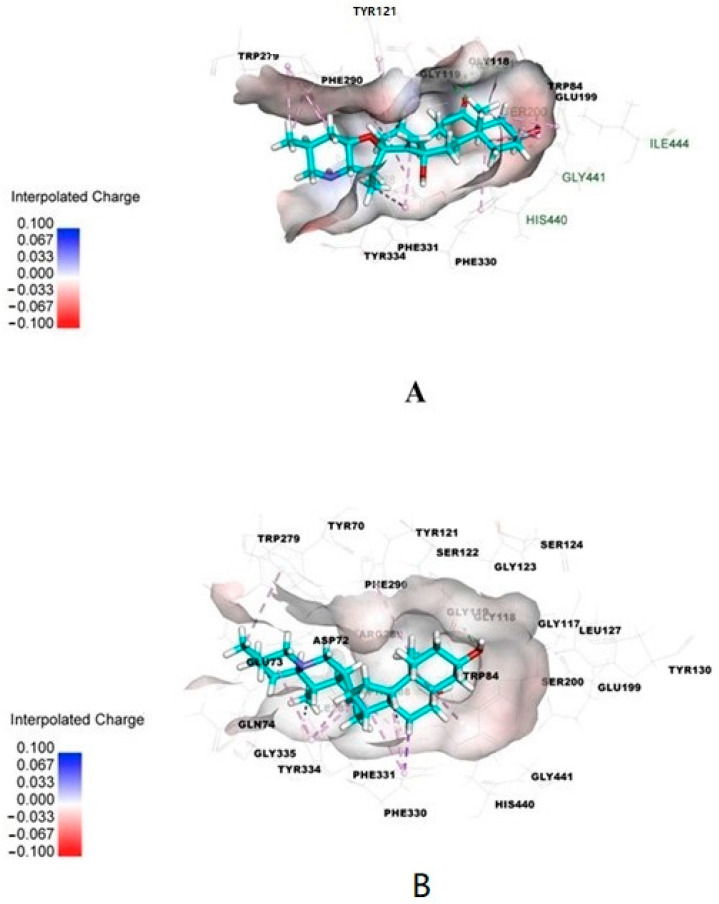
Molecular docking results: (**A**) walujewine (cyanic sticks) in complex with acetylcholinesterase (gray fragments); (**B**) tortifoline (cyanic sticks) in complex with acetylcholinesterase (gray fragments). Reprinted with permission from Ref. [28]. Copyright 2021 Elsevier.

Isosteroidal alkaloids isolated from *Fritillaria walujewii* were evaluated for acetylcholinesterase inhibiting activity by Ellman’s method and by molecular docking [7]. The investigated alkaloids exhibited various inhibition activity with IC_50_ values from 5.85 μM for tortifoline to 93.99 μM for peimisine. In the same experiments, for galantamine, IC_50_ = 2.80 μM. For the most selective compound, walujewine, and most active compound, tortifoline, molecular docking calculations were also performed. The kinetic analysis showed that walujewine A, B and C, tortifoline, a sinpeinine A, hepehenizioiside and walujewine E were mixed-type reversible inhibitors of acetylcholinesterase, simultaneously binding to the catalytic and peripheral anionic sites, which was verified by in silico docking studies [7]. The results obtained for molecular docking are presented in Figure 3. Alkaloids from *Fritillaria walujewii* bulbs showed significant in vitro activity against butyrylcholinesterase [7]. For eight alkaloids, the obtained IC_50_ values were lower than IC_50_ obtained in the same conditions for galantamine. The lowest IC_50_ value was obtained for tortifoline (2.08 μM). Other alkaloids walujewine C and sinpeinine A exhibited high activity with IC_50_ = 2.58 μM and 3.05 μM, respectively. The obtained results indicate the potential of these alkaloids as butyrylcholinesterase inhibitors. The molecular docking simulation also showed that the active compounds tortifoline and sinpeinine A showed many interactions with the catalytic active site and peripheral anionic site gorges of butyrylcholinesterase, indicating a mixed-type inhibition. Absorption, distribution, metabolism, excretion and toxicity (ADMET) analysis was also performed for the investigated alkaloids. Walujewine A, tortifoline and sinpeinine A showed high intestinal absorption, but only tortifoline and sinpeinine A well penetrated the blood–brain barrier. These alkaloids also are nonhepatotoxic and are not CYP2D6 inhibitors. Based on the investigations, the two alkaloids from *Fritillaria walujewii* tortifoline and sinpeinine A can be selected as candidates for further in vivo investigations as compounds potentially useful in the therapy of neurodegenerative diseases.

In the search for potent acetylcholinesterase inhibitors, a group of researchers tested steroidal alkaloids isolated from *Holarrhena pubescens* barks [42]. The in vitro determination of anti-acetylcholinesterase activity was performed by thin-layer chromatography and spectrophotometric methods. Four alkaloids, mokluangin A, B, C and antidysentericine, exhibit the highest acetylcholinesterase inhibition activity with IC_50_ values ranging from 1.44 to 4.09 μM. The IC_50_ value obtained in the experiments for galantamine was equal to 0.51 μM. The results obtained by the molecular docking calculations demonstrated that all compounds can bind at the aromatic gorge of acetylcholinesterase with estimated binding free energies, which correlated well with the in vitro inhibitory profiles, and hydrophobic and hydrogen bonding interactions contribute mainly to the binding of the alkaloids where the substituents at C-3 are a crucial functional group for the acetylcholinesterase activity inhibition.

The anti-acetylcholinesterase potential of piperidine alkaloids from Senna spectabilis was tested using modified Ellman method and TLC [43]. TLC was performed on silica gel plates with mobile phase containing chloroform, methanol and ammonia. After separation, the plates were sprayed with the enzyme solution, thoroughly dried, and incubated at 37 °C for 20 min. Enzyme activity was detected by spraying with a solution consisting of 1-naphthyl acetate in ethanol with aqueous Fast Blue B salt solution. Acetylcholinesterase inhibitors appeared as clear zones on a purple-colored background. IC_50_ values obtained by the spectrophotometric method were 0.29 μg/mL for (−)-cassine, 0.52 μg/mL for (−)-spectaline, 5.85 μg/mL for (−)-3-O-acetylcassine and 12.01 μg/mL for (−)-3-O-acetylspectaline [43]. The IC_50_ values obtained for some investigated alkaloids were lower than or comparable to those obtained for physostigmine used as a positive control (IC_50_ = 0.51 μg mL^−1^). The authors concluded that the alkyl side chain significantly contributed to the anti-acetylcholinesterase potency of the alkaloid, and that the compounds with shorter alkyl side-chain were more active than the homologous alkaloids.

One of the most commonly used drugs for the treatment of neurodegenerative diseases is Amaryllidaceae alkaloid galantamine. For this reason, many Authors evaluated other alkaloids from this family of plants. QSAR analysis and molecular docking were applied for evaluation of Amaryllidaceae alkaloids as inhibitors of human acetylcholinesterase activity [13]. Four qualitative QSAR models based on random forests were developed to evaluate alkaloid inhibitors and noninhibitors of human acetylcholinesterase from Amaryllidaceae alkaloids. The models have a wide range of applicability and allowed the interpretation of 23 descriptors responsible of the inhibition of human acetylcholinesterase. In silico investigations showed the most promising acetylcholinesterase inhibition activity of 11 alkaloids from the Amaryllidaceae family. From these alkaloids, two were recommended as candidates for further investigation: lycoramine, which showed the highest inhibitory activity, and masonine, which presented interactions in the catalytic anionic site and peripheral anionic site. 

**Figure 4:**
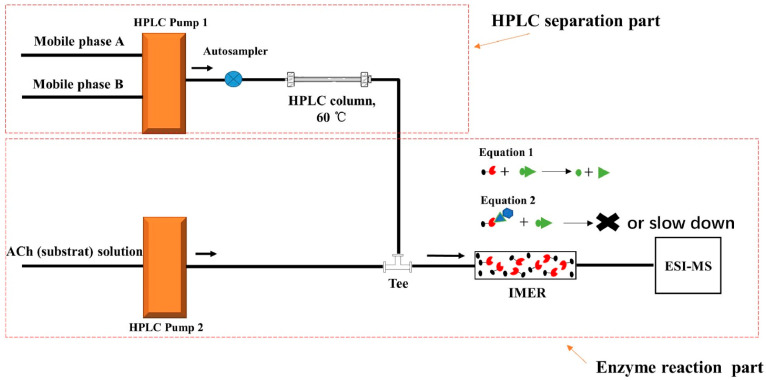
HPLC-IMER-MS bioanalytical device. Immobilized AChE ((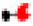) continuously converts ACh (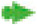) into Cho (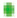) when no AChE inhibitors are eluted from the HPLC column (Equation (1)); when AChE inhibitors (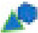) are present in the eluate, they bind to the immobilized AChE, resulting in an increase in remaining ACh and a decrease in detected Cho (Equation (2)). Reprinted with permission from Ref. [25]. Copyright 2017 Elsevier.

Online acetylcholinesterase activity inhibition determination by high-performance liquid chromatography–mass spectrometry hyphenated with an immobilized enzyme reactor was also proposed [25]. The scheme of the system is presented in Figure 4. This proposed bioanalytical device allows a qualitative comparison of the inhibitory strengths of acetylcholinesterase inhibitors by the corresponding acetylcholine peak areas (mass signal) obtained after a chromatographic separation and the elution through the immobilized enzyme reactor. The authors applied the bioanalytical device for the determination of acetylcholinesterase inhibition activity of the extract obtained from Lycoris radiata containing known acetylcholinesterase inhibitors such as alkaloids galanthamine, lycoramine and dihydro-latifaliumin C, for which, previously, a cholinesterase inhibition activity was determined.

**Figure 5:**
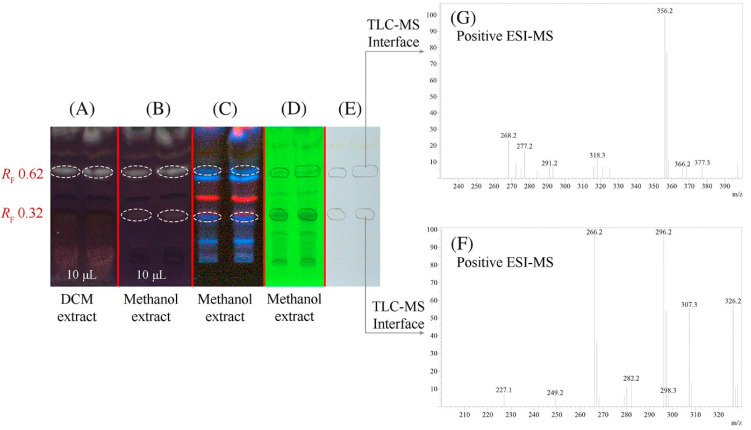
HPTLC chromatograms of methanolic and dichloromethane (DCM) extracts from cherimoya peel on silica gel 60 F_254_ plates using a mobile phase composed of chloroform/methanol/ethyl acetate (80:14:6, *v*/*v*/*v*). HPTLC-acetylcholinesterase bioassay photo-documented under white light of dichloromethane (**A**) and methanolic extract (**B**); photo-documentation at 366 nm-fluorescence (**C**) and 254 nm-UV (**D**); selected bands marked with soft pencil for elution to MS via TLC-MS interface (**E**) and ESI-MS spectra of selected bands (**F**,**G**). Reprinted with permission from Ref. [44]. Copyright 2019 Wiley.

Various another isoquinoline alkaloids have been tested as potentially acetylcholinesterase inhibitors. The anti-acetylcholinesterase activity of the alkaloids anonaine, glaucine and xylopine from Annona cherimola was evaluated using high-performance thin-layer chromatography coupled with mass spectrometry (HPTLC-MS) [44]. Chromatography was carried out on silica gel plates with a mobile phase that contains the enzyme substrate 1-naphthyl acetate. After separation, the mobile phase was removed, and an enzymatic solution in buffer at pH 7.8 was sprayed on a TLC plate. The liquid excess was quickly removed but not completely in order to keep the enzyme active. Next, the plate was incubated at 37 °C for 10 min. Immediately, a Fast Blue B salt aqueous solution was sprayed onto the plate to obtain a purple background, which contrasts with colorless inhibition zones. Acetylcholinesterase inhibitors were identified by direct analysis by means of a TLC-MS interface [43]. HPTLC chromatograms and mass spectra are presented in Figure 5. In the experiments, donepezil was used as a positive control. Anonaine, glaucine and less xylopine showed anti-acetylcholinesterase activity. 

Acetylcholinesterase activity inhibition by some isoquinoline alkaloids and Sanguinaria Canadensis extract was determined using the HPLC-DAD method [45]. Most investigated alkaloids and all Sanguinaria canadensis extracts exhibited acetylcholinesterase activity inhibition. IC_50_ values obtained for alkaloid standards were from 0.36 for berberine to 23.13 μg/mL for protopine and from 61.24 to 89.14 μg/mL for Sanguinaria canadensis extracts collected before, during and after flowering. The synergistic effect for mixtures of the investigated alkaloids was also investigated. For all combinations of alkaloid pairs, the inhibition of acetylcholinesterase activity was higher than the activity of single alkaloids at the same concentrations. The strongest synergy effect was observed for the mixture containing all investigated alkaloids.

Butyrylcholinesterase inhibition activity of Aristotelia serrata leaf extract containing alkaloids aristoteline, aristoserratine, aristotelinone, serratenone, makomakine, aristoserratenine, aristomakine, serratoline, isohobartine, aristomakinine, isosorelline and tasmanine was investigated using Ellman’s method [46]. The IC_50_ concentrations determined by the method for the investigated extract was 26.2 μg/mL.

Other examples of the determination of acetylcholinesterase and butyrylcholinesterase activity inhibition and antioxidant activity by alkaloids from various plant extracts are presented in Table 1, Table 2 and Table 3.

### 2.2. Essential Oils

Essential oils are mixtures of bioactive compounds that are synthesized by plants as secondary metabolites, such as terpenes and terpenoids. The compositions of essential oils are not only different in various plant species but can also be influenced by environmental factors such as soil type, climate and harvest time. In the literature, plants rich in essential oils have been proven to be a potential source of active acetylcholinesterase and butyrylcholinesterase inhibitory compounds, mainly because of the presence of monoterpenic and sesquiterpene hydrocarbons, oxygenated sesquiterpenes, and phenylpropanoids [20].

Most often, the acetylcholinesterase inhibitory properties of various essential oils are determined using the spectrophotometric method proposed by Ellman with the application of a 96-well microplate. 

The acetylcholinesterase inhibition activity of essential oils from Piper betle L. leaves was tested using the modified spectrophotometric method [47]. Essential oils of all the varieties, except Chhaanchi, significantly inhibited the acetylcholinesterase activity in a dose-dependent manner with IC_50_ from 0.10 μg/mL for var. Ghanagete to 0.47 μg/mL for var. Bagerhati [47]. Authors compared the chemical composition of investigated essential oils with anti-acetylcholinesterase activity. The essential oils obtained from betel leaves had much higher acetylcholinesterase inhibitory activity than the individual components, which suggests the synergism of their action. 

De Oliveira et al. investigated the acetylcholinesterase inhibition activity of Piper divaricatum leaves essential oil obtained by supercritical CO_2_ extraction [48]. The determination of the extract inhibition activity was performed by the application of the TLC method. In the procedure, after loading essential oils, TLC plates were sprayed with acetylcholinesterase solution, dried and incubated at 37 °C for 20 min. The enzymatic activity was detected by spraying with a solution of 1-naphthyl acetate in ethanol and aqueous solution of fast blue B salt. Based on the molecular docking, the authors found that β-elemene, eugenol, eugenyl acetate and methyl eugenol are capable of interacting with different residues belonging to the active site of acetylcholinesterase, such as His447 [48]. The results of perresidue free energy decomposition demonstrated that the molecules, during the simulation, performed interactions with residues of the active site that are important for the enzymatic activity inhibition. 

**Figure 6:**
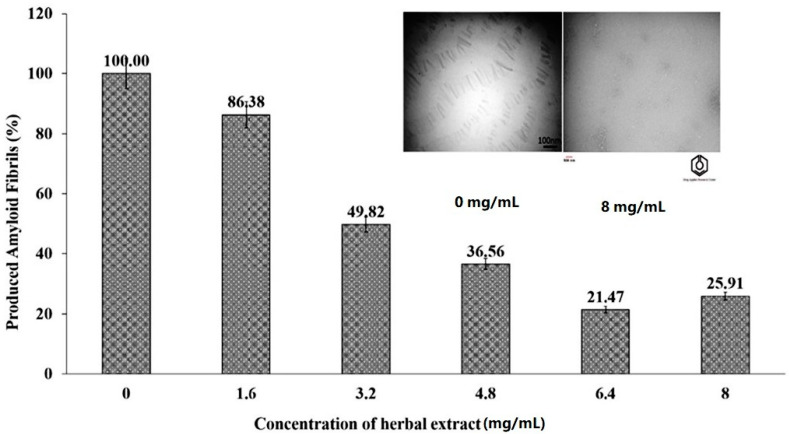
Produced amyloid fibrils (%) at different concentrations (0–8 mg/mL) of *Citrus aurantium* extract. Inhibition of amyloid fibrils is completely obvious in electron micrographs. Reprinted with permission from Ref. [19]. Copyright 2019 Elsevier.

The extract obtained from blossoms of Citrus aurantium containing essential oils exhibited anti-acetylcholinesterase, inhibitory properties on the production of amyloid nanobiofibrils and antioxidant activity. The extract showed acetylcholinesterase inhibition with IC_50_ = 42.8 mg/mL [19]. The inhibitory effects of fragrant essential oils obtained from Citrus aurantium on the production of amyloid nanobiofibrils from bovine serum albumin was also investigated [19]. The authors proposed that the possible mechanism of the extract action is based on the inhibition of protein aggregation via the disruption of π-stacking interactions between aromatic protein residues by aromatic molecules that are active components of the extract. The percentages of produced amyloid fibrils at different concentrations of the extract obtained from blossoms of Citrus aurantium are presented in Figure 6. Citrus aurantium extract has an antioxidant activity and entrapped 94.25% of the available free radicals at the concentration of 8 mg/mL of the extract [19]. The activity was determined using a DPPH assay. Due to its multidirectional activity, the extract obtained from blossoms of Citrus aurantium may be an interesting candidate for further investigations.

The multitargeted screening of anti-neurodegenerative disease potential was performed for *Angelica purpurascens* (Avé-Lall.) Gilli. fruit and roots extracts. The cholinesterase inhibition activity of essential oil from the extracts was detected using Ellman’s method [49]. The dichloromethane fraction of fruit exhibited the highest anti-acetylcholinesterase activity (39.86% inhibition at the concentration 20 mg/mL). Molecular docking for the most active compound oxypeucedanin was also performed. The butyrylcholinesterase inhibition activity of essential oils from Angelica purpurascens (Avé-Lall.) Gilli. was also investigated using Ellman’s method [49]. The highest inhibition activity was determined for fruit hexane fraction (84.02% inhibition at the concentration 20 mg/mL). For the most active compound, oxypeucedanin molecular docking was also performed. Oxypeucedanin exhibited a high dock score against acetylcholinesterase (1EVE) with −7.523 kcal/mol and for 1P0I with −4.232 kcal/mol. The antioxidant activity of essential oils from Angelica purpurascens (Avé-Lall.) Gilli. was examined by DPPH radical scavenging assay [49]. The highest antioxidant activity showed extracts obtained from roots and fruits.

Essential oils obtained from the leaves of Cymbopogon flexuosus, the flowers of Pelargonium x ssp and the resin oil of *Copaifera officinalis* were evaluated by modified Ellman method in terms of their acetylcholinesterase inhibition activity [18]. The IC_50_ values obtained for the investigated essential oils were from 11.92 to 28.18 μg/mL. The increase in the biological activities of these essential oils, through their sustained release obtained by encapsulating them in chitosan microparticles, was also investigated. The obtained results indicated that the microparticles loaded with essential oil presented better acetylcholinesterase inhibition activity when used over a longer period of time, with results comparable to those of free oil. In toxicological tests, Artemia salina larvae assays were used. Samples with an LC_50_ of above 1000 μg·mL^−1^ are considered nontoxic, and samples with an LC_50_ of below 100 μg·mL^−1^ are considered highly toxic. *Cymbopogon flexuosus* oil and copaiba (*Copaifera officinalis*) resin oleo showed low toxicity in the test, while copaiba essential oil and geranium (Pelargonium × ssp) essential oil showed high toxicity and a greater inhibition of acetylcholinesterase, indicating a potential bioactivity for neurodegenerative diseases. 

**Figure 7:**
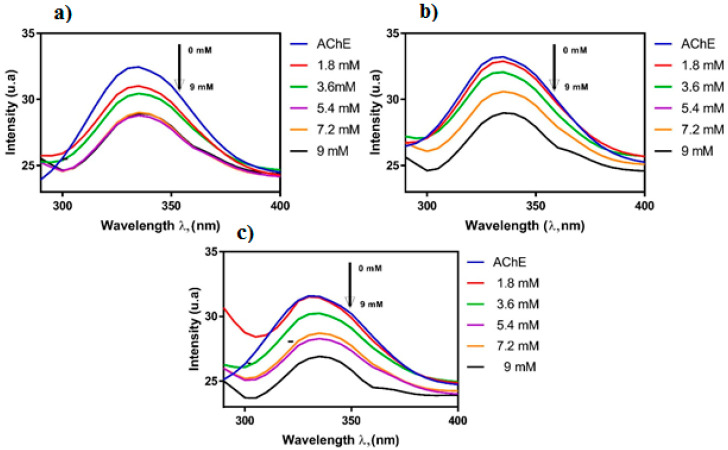
Fluorescence suppression spectra of AChE at 25 °C (**a**), 30 °C (**b**) and 35 °C (**c**) (kexc. = 290 nm), in 0.5 mol/L Tris-HCl buffer pH 8.0, in presence of S. guianensis essential oil. The enzyme from E. electricus was kept in a concentration of 0.67 mg/mL, and the essential oil was titrated separately (as background to subtract) at concentrations from 1.8 to 9 mmol/L. Reprinted with permission from Ref. [50]. Copyright 2021 Elsevier.

**Figure 8:**
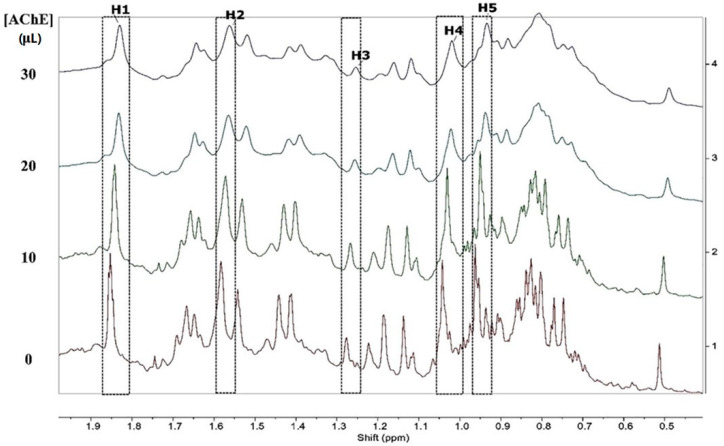
1H RMN titration of essential oil of S. guianensis with AChE (0.67 mg/mL) in DMSO d6. Titrations were performed with AChE of 0 (red line), 10 (green line), 20 (blue line) and 30 uL (purple line) [49].

Another group of researchers described investigations on the interaction between the essential oil of *Siparuna guianensis* Aubl. and acetylcholinesterase activity in order to obtain a new biologically active molecule capable of being used for the treatment of Alzheimer’s disease [50]. The mechanism of inhibition was investigated by spectrofluorimetric interactions between the essential oil and the enzyme, 1H NMR titration and molecular docking. The titration technique by fluorescence quenching is widely used to assess the interactions between ligands and proteins. The essential oil was titrated in a solution containing the enzyme acetylcholinesterase, and the fluorescence spectrum showed a decrease in its intensity, indicating quenching caused by the inhibitor (Figure 7). The reduction in the fluorescence intensity is described by the Stern–Volmer equation, where the ratio between the maximum fluorescence intensities of protein without and with the quencher is used, for each concentration of essential oil. Using a log–log plot, it was possible to obtain the constants related to the quenching of the enzyme in the presence of the essential oil. The results obtained by the titration technique by fluorescence quenching suggest that the binding process was mainly exothermic with favorable enthalpy, and hydrogen bonds and van der Waals forces played a key role in the binding. The 1H NMR spectrum can be a useful tool to analyze the hydrogen atoms involved in the interactions between substances and biomolecules. After the titration of acetylcholinesterase by the inhibitor solution took place, a gradual shift in the shyobunone/derivative peaks toward the high field occurred, i.e., hydrogen shielding of the compound occurs during contact with the enzyme. This indicates an increase in electron density in the compound hydrogens, which may mean that hydrogen bonds are involved in the interaction between acetylcholinesterase and the inhibitor [50]. The 1H NMR spectrum and the indication of hydrogen atoms participating in the interaction are shown in Figure 8. 

Essential oil from aerial parts of *Clinopodium brownie*, which contained as main components pulegone, menthone and β-acorenol, was evaluated as a potential butyrylcholinesterase inhibitor using Ellman’s method [26]. In the investigation, the essential oil showed selective inhibitory activity for butyrylcholinesterase with an IC_50_ = 13.4 μg/mL, while it was weakly active against acetylcholinesterase with IC_50_ > 250 μg/mL [26].

### 2.3. Phenolic Compounds

Plant phenols, commonly referred to as polyphenols or biophenols, have several biological activities such as antioxidant and neuroprotective effects and can be used in the prevention and/or management of diabetes and obesity. Phenolic compounds are well-acknowledged as potential metal chelation agents and inhibitors of lipid peroxidation. Various phenolic compounds were tested for acetylcholinesterase activity inhibition properties, most often simultaneously with antioxidant activity investigations. Among all compounds of plant origin, this group of compounds was most often tested for the activity of inhibiting acetylcholinesterase. More recently, various phenolic compounds obtained from different plant extracts have been investigated for their acetylcholinesterase inhibitory activity. Especially, plants exhibiting multidirectional therapeutic action are especially promising candidates for the treatment of neurodegenerative diseases.

For example, *Pulmonaria officinalis* and *Centarium umbellatum* extracts containing polyphenols, flavones and proanthocyanidins showed multitargeted activity against acetylcholinesterase and tyrosinase and possessed antioxidant properties [34]. The extracts were evaluated as acetylcholinesterase activity inhibitors by the spectrophotometric Ellman method. The ethanolic extracts from both plants exhibit significant acetylcholinesterase inhibitory activity. The inhibition values were >70% for the samples at a concentration of 3 mg/mL. The highest inhibition value was obtained for the *Centarium umbellatum* ethanolic extract (94.24% inhibition) and corresponded to the presence of high contents of flavone. The tyrosinase inhibitory potential of Pulmonaria officinalis and *Centarium umbellatum* extracts was also tested. The tyrosinase activity was spectrophotometrically measured using 3-(3,4-dihydroxyphenyl)-Lalanine (L-DOPA) as a substrate. Tyrosinase aqueous solution, plant extract and phosphate buffer at pH 7.0 were mixed and incubated for 15 min at 30 °C. Next, L-DOPA was added, and the absorbance at 475 nm was measured for 3 min at 475 nm. The same reaction mixture with the plant extract replaced by an equivalent amount of phosphate buffer served as a blank [34]. An extract from *Centarium umbellatum* at a concentration of 3 mg/mL inhibited 74.39% of tyrosinase activity. The antioxidant activity of *Pulmonaria officinalis* and *Centarium umbellatum* extracts was evaluated using a DPPH assay and reducing power [34]. The investigated extracts showed high antioxidant activities. Pulmonaria officinalis and Centarium umbellatum extracts at concentrations of 3 mg/mL also inhibited 84.9 and 75.47% DPPH radical scavenging activity, respectively [34].

An extract from *Tithonia diversifolia* (Hemsl.) A. rich in phenolic acids (gallic acid, chlorogenic acid, caffeic acid and p-coumaric acid) and flavonoids (apigenin) exhibited anti-cholinesterase activity with IC_50_ = 39.27 μg/mL [37]. The obtained results suggest that *Tithonia diversifolia* leaf has potential application as acetylcholinesterase activity inhibitor, exhibiting a stronger activity than the standard drug prostigmine (IC_50_ = 50.02 μg/mL). The potential mechanism of the neuroprotective properties may be based on inhibiting cholinesterase activities and thwarting oxidative-stress-induced neurodegeneration. The extract also showed butyrylcholinesterase inhibition activity with IC_50_ = 35.01 μg/mL [37]. The anti-butyrylocholinesterase activity of the extract was also higher than the activity of the standard drug prostigmine (IC_50_ = 48.56 μg/mL). The extract obtained from *Tithonia diversifolia* additionally exhibited antioxidant activity determined by DPPH radical scavenging abilities with IC_50_ = 41.05 μg/mL, ABTS acid radical scavenging with IC_50_ = 33.51 μg/mL and iron chelation with IC_50_ = 38.50 μg/mL [37].

An extract obtained from the leaves of Antidesma *madagascariense* containing flavonoids and other phenolic compounds showed acetylcholinesterase inhibition activity with IC_50_ = 35.97 μg/mL [51]. The investigated extract was the most potent inhibitor of acetylcholinesterase compared to its fractions (IC_50_ from 289.9 to 492.6 μg/mL), but its activity was significantly lower than the positive control, galanthamine, with an IC_50_ = 3.58 μg/mL. The extract was also evaluated as an antioxidant using ABTS radical scavenging assay, DPPH radical scavenging assay and ferric reducing antioxidant power assay [51]. Three fractions of the extract exhibited very high antioxidant properties with IC_50_ values determined by DPPH radical scavenging assay from 1.26 to 1.61 μg/mL, which were significantly lower than the positive control, ascorbic acid (IC_50_ = 5.89 μg/mL). In the investigations, the cytotoxicity of the extracts was tested against Vero cells isolated from kidney epithelial cells extracted from an African green monkey. Acetone extract and hexane fraction were noncytotoxic with IC_50_ = 201.85 and 195.3 μg/mL, respectively, while decoction showed a cytotoxic effect (IC_50_ = 4.15 μg/mL), but the cytotoxicity was lower than Actinomycin D used as the positive control (IC_50_ = 0.05 μg/mL).

Ozkan et al. evaluated the anti-acetylcholinesterase activity of *Hypericum neurocalycinum* and *Hypericum malatyanum* containing active components such as two pseudohypericin, hypericin, chlorogenic acid, rutin, hyperoside, isoquercitrin, kaempferol, quercitrin, quercetin, amentoflavone and hyperforin using the spectroscopic Ellman method [38]. Hypericum neurocalycinum and *Hypericum malatyanum* extracts at concentrations of 10 mg/mL inhibited acetylcholinesterase activity in 85.78 and 62.24%, respectively. The antioxidant activity of *Hypericum neurocalycinum* and *Hypericum malatyanum* extracts was estimated using four methods, three of them based on the evaluation of the free radical scavenging activity (the inhibition of lipid peroxidation, DPPH radical scavenging activity and superoxide radical scavenging activity) and one based on measuring their iron-reducing capacity [38]. Based on HPLC-DAD analysis, the presence of two naphthodianthrones (pseudohypericin and hypericin), chlorogenic acid, rutin, hyperoside, isoquercitrin, kaempferol, quercitrin, quercetin, amentoflavone and hyperforin were found as main compounds in the methanol extracts. *Hypericum neurocalycinum* exhibited stronger antioxidant properties than Hypericum malatyanum due to a higher activity on scavenging DPPH and superoxide anion radicals, and an inhibition of lipid peroxidation, which corresponded to higher amounts of antioxidant compounds (flavonoids) such as rutin, quercetin and kaempferol.

Acetylcholinesterase inhibitory activities of extracts obtained from leaves and stem barks of *Macaranga hurifolia* Beille, *Sterculia tragacantha* Lindl. and *Zanthoxylum gilletii* were investigated using a modified Ellman method [52]. The highest acetylcholinesterase inhibition activity exhibited extract from *Macaranga hurifolia*. The antioxidant properties of these extracts were spectrophotometrically screened by different experiments as phosphomolybdenum, quenching of radicals (DPPH and ABTS), reduction potentials (FRAP and CUPRAC) and ferrous ion chelating [52]. An extract obtained from *Sterculia tragacantha* had the highest antioxidant activity. Radical scavenging assays also showed that the stem barks of all three investigated plants were better scavengers than leaf extracts.

Li et al. tested (−)-Epicatechin gallate, 1,2,3,4,6-O-pentagalloylglucose, rhodionin, herbacetin and rho-diosin isolated from the root of *Rhodiola crenulata* as acetylcholinesterase inhibitors [53]. The investigated compounds exhibited dose-dependent inhibitory effects with IC_50_ ranged from 57.50 to 2.43 μg/mL. The investigations were performed using molecular docking and isothermal titration calorimetry methods. The docking results showed that the binding energies of most compounds indicated that these compounds could inhibit the acetylcholinesterase by binding into the ligand pock. Isothermal titration calorimetry is a sensitive research tool for examining the binding interactions where the differential enthalpy during the binding process is monitored. The results obtained by both methods indicated that the binding mechanism of these active compounds into the structure of acetylcholinesterase is based on electrostatic interaction and hydrogen bonding [53].

Other candidates for further investigations for the use of treating neurodegenerative diseases are extracts and isolated biologically active compounds from Artemisia annua. Active compounds: scopoletin, chrysosplenetin, eupatin and 3-O-β-d-glucopyranoside of sitosterol isolated from aerial parts of Artemisia annua were tested as potential acetylcholinesterase inhibitors [54]. The determination of anti-cholinesterase inhibition activity was performed by the Ellman spectrophotometric method. The crude extract inhibited acetylcholinesterase activity with an IC_50_ value of 87.43 μg/mL. Artemisinin and chrysosplenetin had the highest anti-acetylcholinesterase activity from the investigated compounds, with IC_50_ values of 29.34 and 27.14 μg/mL, respectively. The anti-inflammatory activity of Artemisia annua crude extract, extract fractions and active compounds isolated from the extract was evaluated by the determination of nitric oxide inhibitory activity [54]. Investigations were performed on mouse macrophages (RAW 264.7) cell line, which were cultured in a 96-well microtitre plate with activated lipopolysaccharides. The amount of nitric oxide released was determined by the Griess method. At the lowest concentration tested (6.25 μg/mL), the crude extract and one fraction had the highest NO inhibitory activity (72.39 and 71.00% inhibition, respectively) without significant toxicity on the viability of macrophage cells (93.86 and 79.87% of cell viability, respectively) [54].

The anti-acetylcholinesterase activity of extracts obtained from the roots of *Lepisorus mehrae*, the leaves of *Pleurospermum benthamii*, and the rhizomes of *Roscoea auriculata* was tested using a modified Ellman method [55]. The authors reported that the highest inhibitory potential against acetylcholinesterase was observed for the hexane extract of *Roscoea auriculata,* with an IC_50_ value of 0.260 mg/mL. IC_50_ = 0.001 mg/mL for galantamine used as reference in the experiment. The anti-butyrylcholinesterase activity of these extracts contained was also evaluated using a modified Ellman method [55]. The highest butyrylcholinesterase activity inhibition was observed for ethyl acetate extract obtained from *Pleurospermum benthamii*, with an IC_50_ value of 0.077 mg/mL (IC_50_ = 0.026 mg/mL for galantamine). The inhibition of tyrosinase activity by *Lepisorus mehrae*, *Pleurospermum benthamii*, and *Roscoea auriculata* extracts was also examined [55]. The inhibition of tyrosinase was determined using the modified dopachrome method. To plant extracts of different concentrations, a potassium phosphate buffer at pH 6.5, mushroom tyrosinase (1000 U/mL), was added. The mixture was pre-incubated at 27 °C for 10 min, followed by the addition of 5 mM l-3,4-dihydroxyphenylalanine (L-DOPA). The absorbance was measured at 492 nm in a UV–visible spectrophotometer. The highest tyrosinase inhibition activity was exhibited by a methanol extract from *Pleurospermum benthamii* with an IC_50_ value of 0.792 mg/mL. In the investigations of the reference compound kojic acid, IC_50_ = 0.018 mg/mL. The antioxidant property of investigated extracts was evaluated using a DPPH radical scavenging assay and ABTS radical scavenging assay [55]. The highest antioxidant activity was found by both DPPH and ABTS assays for aqueous extract from Lepisorus mehrae with IC_50_ values 32.45 and 9.70 μg/mL, respectively. For gallic acid used in the investigations as reference compound by DPPH and ABTS assays, IC_50_ = 5.12 and 1.96 mg/mL, respectively. Especially interesting is the inhibition of butyrylcholinesterase activity by ethyl acetate and water extracts from *Pleurospermum benthamii*, which is comparable with the inhibition activity of galantamine.

The acetylcholinesterase inhibition activity of flavonoids isolated from the leaves of *Polygonum limbatum*, twigs of *Dorstenia barteri*, aerial parts of *Dorstenia mannii* and twigs of *Dorstenia dinklagei* were tested by the Ellman method [56]. The investigated flavonoids showed anti-cholinesterase activity with IC_50_ from 5.93 μg/mL obtained for the most active compound isobavachalcone, to 8.76 μg/mL obtained for 6-prenylapigenin. For eserine sed as standard, IC_50_ = 4.94 μg/mL. For the determination of the anti-inflammatory activity of flavonoids isolated from *Polygonum limbatum* and Dorstenia species, the soybean lipoxygenase inhibition assay was used, as well as determining the amount of nitric oxide released from RAW 264.7 murine macrophage cells [56]. For the most active compound isobavachalcone, IC_50_ = 25.92 μg/mL in a soybean lipoxygenase inhibition assay, and this was similar to the IC_50_ value obtained for quercetin used as standard (IC_50_ = 25.53 μg/mL). The tested flavonoids also showed no significant cytotoxic effect against macrophages. Based on the results, isobavachalcone can be recommended for further investigation as a promising multipotent agent.

The extract obtained from *Feijoa sellowiana* leaves containing phenolic compounds exhibited acetylcholinesterase activity inhibition with IC_50_ = 120 μg/mL [57]. An enzyme inhibition kinetic investigation was also performed. The obtained results showed that components of *Feijoa sellowiana* extract affected the reaction velocity catalyzed by acetylcholinesterase but without the modification of the Michaelis constant value, which recalls the behavior of noncompetitive inhibition. The pancreatic lipase activity inhibition by *Feijoa sellowiana* leaves extract was evaluated by Mosbah et al. [57]. To evaluate the pancreatic lipase inhibitory activity, *Feijoa sellowiana* leaf extract was dissolved in dimethyl sulfoxide and preincubated for 1 h at room temperature. After preincubation, an aliquot from the reaction mixture was used to estimate the residual pancreatic lipase activity. A control was conducted similarly to the inhibition test, but without the leaves’ extract. The pancreatic lipase activity was measured titrimetrically using a pH-stat at pH 8.5 and 37 °C. Olive oil emulsion was used as a substrate in the presence of sodium deoxycholate. To ensure enzyme adsorption at the lipid/water interface, purified pancreatic colipase was added to the reaction mixture [57]. The high anti-lipase activity was determined with IC_50_ = 0.3 mg/mL, which is comparable to that of pure tetrahydrolipstatin used as a standard inhibitor against pancreatic lipase.

Bulbs and aerial parts of *Allium nigrum* and *Allium subhirsutum* extracts were tested as a multipotent agent, which can potentially be applied in the treatment of neurodegenerative diseases. These extracts were tested as acetylcholinesterase inhibitors by modified Ellman method [58]. The highest inhibition activity was observed for aerial parts extracts of *Allium nigrum* with IC_50_ values of 6.1 μg/mL. Galanthamine was used as a positive standard, which showed IC_50_ values of 0.106 μg/mL. Investigated extracts were also evaluated as butyrylcholinesterase activity inhibitors [58]. The highest inhibition activity obtained by modified Ellman method was also found for the aerial parts extract of *Allium nigrum* with IC_50_ values of 6.1 μg/mL, while the IC_50_ value obtained for galantamine was equal to 1.04 μg/mL. The authors investigated tyrosinase inhibitory potentials of these extracts [58]. The highest inhibition activity measured by the dopachrome method was observed for the *Allium nigrum* aerial parts extract with IC_50_ values of 22.31 μg/mL. In the same experiments, IC_50_ = 7.9 μg/mL for kojic acid used as standard. A good correlation was observed between the content of phenolic compounds and all tested enzyme inhibition activities.

**Figure 9:**
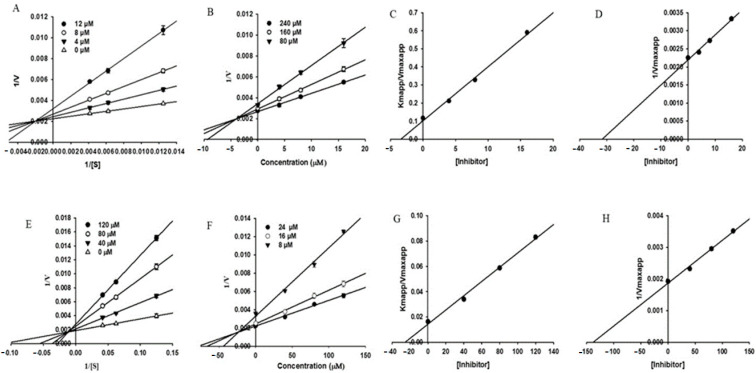
Lineweaver–Burk (**A**,**E**), Dixon (**B**,**F**) and secondary (**C**,**D**,**G**,**H**) plots of glycitein for acetylcholinesterase and butyrylcholinesterase inhibition, respectively. Reprinted with permission from Ref. [59]. Copyright 2021 ACS Publications.

Isoflavone analogs from flowers of *Pueraria lobate* extract was evaluated as acetylcholinesterase inhibitors using modified Ellman method [59]. The IC_50_ value obtained for glycitein, the most active compound isolated from Pueraria lobate, was 143 μM. Kinetic data were graphically presented by the Lineweaver−Burk, Dixon and secondary plots (Figure 9). Glycitein inhibited butyrylcholinesterase activity with IC_50_ = 69.40 μM. Glycitein also inhibited human monoamine oxidases with IC_50_ = 8.30 μM. Inhibition was evaluated in vitro using MAO-Glo chemiluminescent assay kit in a 96-well plate. The results obtained in the kinetics study indicated that glycitein exhibited a mixed mode of inhibition against all the tested enzymes. Molecular docking showed that glycitein can interfere with the activities of enzymes significantly by interacting with orthosteric and allosteric site residues.

Commercial kits were rarely used for evaluation of anti-cholinesterase activity. For example, *Evodia lepta* Merr. roots extract and an extract isolated from bis-coumarins, (±)-dievodialetins A–G were tested as acetylcholinesterase inhibitors using commercial kits [60]. The investigated bis-coumarins exhibited acetylcholinesterase inhibitory activity, with IC_50_ values ranging from 7.3 to 12.1 nM. The neuroprotective effects of the investigated compounds were also investigated using an in vitro cell model (scopolamine, which causes cholinergic nervous system dysfunction). In the MTT assay of scopolamine-treated SH-SY5Y cells; all the compounds exhibited neuroprotective effects. Increased levels of the antioxidant enzyme superoxide dismutase were observed in scopolamine-treated SH-SY5Y cells. Active compounds isolated from *Evodia lepta* increased the activity of antioxidant enzyme superoxide dismutase. In addition, these compounds significantly decreased the levels of the IL-1β and IL-6, which are key mediators of the inflammatory response. Due to the multifunctional effect of the tested compounds, they could be indicated for further investigations.

Liu et al. investigated the acetylcholinesterase inhibition activity of isoflavane glycosides from stems of *Medicago sativa* L. using a modified Ellman method with the application of a 96-well microplate [61]. Three of the investigated compounds showed significant anti-acetylcholinesterase activity, with IC_50_ values between 31.13 and 43.32 μg/mL. An eventual mechanism of interaction between the active compounds and acetylcholinesterase was performed by molecular docking studies. The obtained results indicated that the amide residues His 447 and Glu 202 could be considered key active residues interacting with natural ligands. Most active compounds exhibited statistically potent neuroprotective effects against H_2_O_2_-induced human neuroblastoma (SH-SY5Y) cell death compared with the H_2_O_2_-treated group [60]. For the determination of antioxidant activity of isoflavane glycosides from *Medicago sativa* L., an ABTS radical scavenging assay, DPPH radical scavenging assay and ferric reducing antioxidant power assay were performed [61]. The highest antioxidant properties were found for (3R)-7,5′-dihydroxy-2′,3′,4′-trimethoxy-isoflavane-5′-O-β-D-glucoside with IC_50_ = 19.54 μg/mL (obtained by DPPH radical scavenging assay) and 20.74 μg/mL (obtained by ABTS radical scavenging assay). In both tests, the IC_50_ for the isoflavane glycoside were lower than those obtained for trolox used as positive control. 

The ability to inhibit acetylcholinesterase and butyrylcholinesterase by six diarylheptanoids and two flavonoids derived from rhizomes of *Alpinia officinarum* were evaluated using the Ellman method and molecular docking [62]. Compounds isolated from the plant exhibited IC_50_ values from 2.6 to 87.3 μM against acetylcholinesterase activity and from 35.2 to 70.7 μM against butyrylcholinesterase. For tacrine used as a positive control, IC_50_ = 111.8 and 8.9 μM against acetylcholinesterase and butyrylcholinesterase, respectively. The nature of the interaction between investigated compounds isolated from the plant and acetylcholinesterase was revealed using enzyme kinetic studies. Based on the obtained results, authors found that these inhibitors interact with enzyme by competitively binding to the active site of acetylcholinesterase. Molecular dynamics studies demonstrated stable binding energies and the interactions between enzyme amino acids and the isolated compound residues.

Flavoalkaloids from Yunnan Black Tea ‘Jin-Ya’ were tested as potent α-glucosidase and acetylcholinesterase inhibitors [63]. Active compounds inhibited acetylcholinesterase with IC_50_ from 10.81 to 34.82 μM.

**Figure 10:**
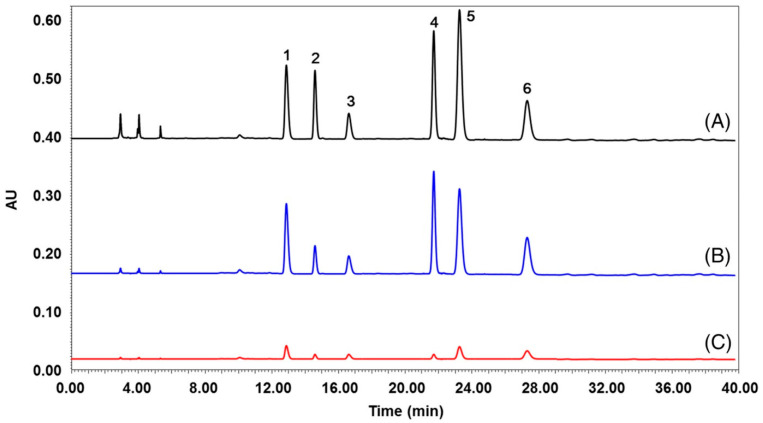
High-performance liquid chromatography profile of the chemical constituents of *Astragalus membranaceus* extract obtained by ultrafiltration. (**A**) *Astragalus membranaceus* extract (the concentration of the extract was the same as that in the ultrafiltration experiment); (**B**) compounds bound to acetylcholinesterase; (**C**) compounds bound to denatured acetylcholinesterase. 1, calycosin-7-O-β-D-glucoside; 2, pratensein-7-O-β-D-glucoside; 3, formononetin-7-O-β-Dglucoside; 4, calycosin; 5, genistein; 6, formononetin. Reprinted with permission from Ref. [64]. Copyright 2021 Wiley.

Calycosin-7-O-β-D-glucoside, pratensein-7-O-β-D-glucoside, formononetin-7-O-β-D-glucoside, calycosin, genistein and formononetin from *Astragalus membranaceus* were tested as acetylcholinesterase inhibitors [64]. In the investigations, ultrafiltration high-performance liquid chromatography-mass spectrometry (UF-HPLC-MS) was applied for the preliminarily screening of enzyme inhibitors based on the specific binding between enzyme and inhibitor from complex extracts of plants. In the ultrafiltration assay, the ligand-bound enzyme complexes are first separated from unbound compounds by ultrafiltration, and then the ligands dissociated from the complexes are potential acetylcholinesterase inhibitors. In Figure 10 are presented the results of the ultrafiltration screening of acetylcholinesterase inhibitors from *Astragalus membranaceus* extract. The profile of the crude extract showed several major peaks (Figure 10A; however, only six peaks were released upon methanol disruption and thus identified as acetylcholinesterase inhibitors (Figure 10B). Denatured acetylcholinesterase showed no or very weak binding to these compounds (Figure 10C). The IC_50_ values against acetylcholinesterase for calycosin-7-O-β-D-glucoside, pratensein-7-O-β-D-glucoside, formononetin-7-O-β-D-glucoside, calycosin, genistein and formononetin were 44.22, 48.09, 49.69, 46.96, 45.13 and 44.83 μg/mL, respectively [64].

Active phenolic compounds from *Citrus limon* peel: neoeriocitrin, isonaringin, naringin, hesperidin, neohesperidin, limonin and poncirin exhibited in vitro acetylcholinesterase inhibition activity with IC_50_ 80.97, 116.45, 81.91, 134.44, 84.69 and 178.13 μmol/L, respectively [64]. In the investigations, ultrafiltration high-performance liquid chromatography–mass spectrometry was also applied for the preliminarily screening of enzyme inhibitors.

Nwidu et al. reported anti-cholinesterase and antioxidant activity of extracts obtained from the leaves, seeds, roots, flowers and bark of *Moringa oleifera* [65]. IC_50_ values for the most potent extracts, root methanolic extract, root ethanolic extract and bark ethanolic extract, were 8.45, 1.75 and 1.73 μg/mL, respectively. The antioxidant properties of *Moringa oleifera* extracts were tested by DPPH radical scavenging assay. For the most active extracts, leaf methanolic extract and food powder methanolic extract, the IC_50_ values were 0.02517 and 0.02579 μg/mL, respectively. 

The inhibition of human monoamine oxidase A and B by flavonoids isolated from aerial parts of *Hypericum afrum* and *Cytisus villosus* was determined by fluorometric kynuramine assay in 384-well plates [66]. The ethyl acetate fractions of *Hypericum afrum* and *Cytisus villosus* extracts showed the highest monoamine oxidase inhibition activity against monoamine oxidase A, with IC_50_ values of 3.37 μg/mL and 13.50 μg/mL, and against monoamine oxidase B, with IC_50_ values of 5.62 and 1.87 μg/mL, respectively. Quercetin, myricetin and chrysin showed the highest monoamine oxidase A inhibitory activity with IC_50_ values of 1.52, 9.93 and 0.25 μM, respectively, while genistein more efficiently inhibited monoamine oxidase B with an IC_50_ value of 0.65 μM. The kinetic study of the inhibition and the investigations of dialysis dissociation of the complex of quercetin and myricetin with the isoenzyme monoamine oxidase A indicated competitive and mixed inhibition, respectively. Both flavonoids exhibited reversible binding to the active site of the enzyme. 

Flavanones and two flavones isolated from the leaves of *Prunus padus var. seoulensis* were tested as monoamine oxidases inhibitors by the spectrophotometric continuous method [67]. The highest selective activity against human monoamine oxidase A and human monoamine oxidase B exhibited rhamnocitrin with IC_50_ = 0.051 μM and 2.97 μM, respectively. The activity of rhamnocitrin was 20.2 times higher than that of the drug toloxatone, with an IC_50_ value of 1.03 μM, 

Extracts obtained from the roots, barks, woods and leaves of *Phyllanthus chamaepeuce* Ridl. were in vitro tested as pancreatic lipase inhibitors. The highest inhibitory activity was exhibited by ethanolic and methanolic leaf extracts, with IC_50_ = 2.98 μg/mL and 4.36 μg/mL, respectively. The results from a kinetic study indicated that ethanolic and methanolic leaf extracts from the plant showed noncompetitive inhibition.

### 2.4. Saponins

Saponins are steroid or triterpenoid glycosides, which exhibit several biological activities, e.g., immunostimulant, hypocholesterolaemic and anticarcinogenic, analgesic, anti-nociceptive, antioxidant, antifungal and antiviral properties. Some compounds belonging to the class of natural compounds have also been tested in terms of acetylcholinesterase activity inhibition. 

The potential of acetylcholinesterase inhibition by saponins and ginsenosides from the stems and leaves of *Panax ginseng* extracts was determined by a modified Ellman method and by ultrafiltration analysis [68]. The samples of extract fractions were incubated in a solution consisting of 10 U/mL acetylcholinesterase and phosphate buffer at pH 7.6 for 0.5 h at 37 °C. After incubation, the binding mixture was filtered through an ultramembrane filter after being centrifuged. The filter was washed three times by centrifugation with phosphate buffer to remove the unbound compositions. The bound compounds were released by methanol. The most active fraction of *Panax ginseng* extract showed anti-acetylcholinesterase activity with IC_50_ = 12.53 μg/mL. IC_50_ values obtained for ginsenosides isolated from the plant were from 4.27 μg/mL for ginsenoside F1 to 20.00 μg/mL for ginsenoside Rc [68].

Polyacetylenes homopanaxynol, homopanaxydol, (9Z)-heptadeca-1, 9-diene-4,6-diyn-3-one and (8E)-octadeca-1,8-diene-4,6-diyn-3,10-diol obtained from *Panax ginseng* were tested as acetylcholinesterase inhibitors using the following procedure [69]. Samples in a dimethylsulfoxide solution at an appropriate concentration were diluted with an assay buffer (50 mM Tris–HCl buffer, pH = 7.8) in a 96-well microplate. An enzyme solution (2.0 U/mL) and solution of 1-naphthyl acetate (18 mM) were added to the mixture and incubated at 37 °C for 1 h. After incubation, 5% sodium dodecyl sulfate solution and Fast Blue B Salt solution (2 mM) were added. The absorbance was measured at 600 nm. The highest antiacetylcholinesterase was determined for (9Z)-Heptadeca-1,9-diene-4,6-diyn-3-one with IC_50_ = 132 µM. The inhibitory activity of polyacetylenes from Panax ginseng were also evaluated as butyrylcholinesterase inhibitors by spectrophotometric method [69]. The raw root hexane extract inhibited a butyrylcholinesterase activity of 20.3% at 100 μg/mL. The inhibitory activities of polyacetylenes obtained from *Panax ginseng* and extracts from root of the plant against β secretase were also investigated [69]. The inhibition activity was evaluated by the following procedure. Samples in a dimethylsulfoxide solution were diluted with an assay buffer (acetate buffer, pH = 4.5 containing 1% triton X-100) in a 96-well microplate. Ten β secretase solution in assay buffer was added to the diluted solution and incubated at 37 °C for 20 min. After incubation, the substrate solution with assay buffer was added and incubated at 37 °C for 2 h. After incubation, the reaction solution was added to a 2.5 M sodium acetate solution to terminate the reaction. The diluted solution was analyzed by HPLC on a C18 column with mobile phase containing acetonitrile, water and formic acid. Detection was performed using a fluorescent detector of excitation at 325 nm and emission at 395 nm. Raw root hexane extract showed the most potent activity of 58.4% inhibition at 250 µg/mL [69].

For the determination of the potential of acetylcholinesterase inhibition by *Panax japonicus* containing saponins, a PC12 cell model and acetylcholinesterase binding were applied [7]. Chikusetsusaponins V, Ib, IV, IVa and IVa ethyl ester were identified as acetylcholinesterase activity inhibitors. In the investigation, the protective effects of *Panax japonicus* leaf extract and its chemical constituent saponins against neuronal damage were determined by pretreating PC12 cells from rat pheochromocytoma with characteristics similar to midbrain dopamine neurons [7].

### 2.5. Other Compounds

Many authors have also tested other compounds of plant origin belonging to various groups for their applicability in neurodegenerative diseases.

The determination of acetylcholinesterase activity inhibition by anthocyanin from blueberry and purple potato extracts was performed by HPLC [24]. The procedure was based on an Ellman reaction performed before HPLC-DAD analysis. The chromatographic analysis was performed on a C18 column thermostated at 37 °C with mobile phase containing methanol–water–triethylamine (40:60:0.05, *v*/*v*/*v*). For the optimization of the experiment conditions, the effect of pH on enzyme activity, reaction temperature on enzyme activity, reaction time on enzyme activity, and acetylthiocholine iodide and acetylcholinesterase concentration on enzyme activity were investigated [24]. The authors recommended the HPLC method especially for the evaluation of the acetylcholinesterase inhibitory activity in samples with deep color.

Lee et al. tested 640 various natural compounds for inhibitory activity against acetylcholinesterase using a modified Ellman method [32]. For the investigated compounds, sargachromanol I and G isolated from the brown alga *Sargassum siliquastrum* and macelignan isolated from *Myristica fragrans* most potently inhibited acetylcholinesterase activity, with IC_50_ values of 0.79, 1.81 and 4.16 μM, respectively. Broussonin A most effectively inhibited butyrylcholinesterase (IC_50_ = 4.16 µM), with IC_50_ values obtained for macelignan, sargachromanol and SCI of 9.69, 10.79 and 13.69 µM, respectively. The kinetics of acetylcholinesterase inhibition by the most active compounds, sargachromanol I and G and macelignan, were also investigated at five different substrate concentrations (0.05, 0.1, 0.2, 0.5 and 1.0 mM) and in the absence or presence of each inhibitor at approximately 1/2 × IC_50_, IC_50_ and 2 × IC_50_ values. Inhibitory patterns and Ki values were determined using Lineweaver–Burk plots and secondary plots. These compounds showed mixed, competitive and noncompetitive inhibition. A docking simulation indicated that the most active compound, sargachromanol I, interacts with acetylcholinesterase at Trp81, while sargachromanol G interacts at Ser119 [32]. 

Lupeol long-chain alkanoic ester, lupeol β-hydroxy fatty acid esters 2c,d (laevigatins I and II) and lupeol and lupeol acetate isolated from the latex of *Periploca laevigata* were tested for acetylcholinesterase inhibition activity [70]. Methanol extract exhibited anti-acetylcholinesterase activity with IC_50_ = 60.90 μg/mL. The highest inhibition activity was observed for lupeol with IC_50_ = 38.31 μg/mL. The results obtained by the authors suggest that the triterpenic skeleton and the free secondary alcohol function at C-3 could be responsible of this activity, while the esterification of the alcohol function may decrease the inhibition of acetylcholinesterase [70].

The acetylcholinesterase inhibition activity determined by modified Ellman method showed that the extract from *Cassia tora* contained triglycerides with IC_50_ = 42.54 mg/mL [30]. The ethyl acetate fraction of *Cassia tora* extract was also evaluated for the potential to prevent amyloidosis by inhibiting the aggregation of monomers and oligomers of the most aggregable amyloid species, β-amyloid 1-42-induced neuronal damage and free radical levels in vitro in human neuroblastoma SK-N-SH and SH-SY5Y cells [30]. The activity was tested using thioflavin-T fluorescence and transmission electron microscopy. The fraction prevented Aβ 1-42 aggregation, inhibited acetylcholinesterase and alleviated Aβ 1-42-induced oxidative stress in human neuroblastoma cells. 

Acetylcholinesterase inhibition by proteases has rarely been investigated. Cysteine protease glycoprotein from the rhizome of *Zingiber montanum* exhibited in vitro acetylcholinesterase activity inhibition [1]. The investigated compound showed profound dose-dependent acetylcholinesterase inhibitory activity with IC_50_ = 1.88 μM. Enzyme kinetic studies were also performed and obtained results indicated on competitive inhibition. Molecular interaction investigations showed that *Zingiber montanum* cysteine protease glycoprotein has binding tendencies with acetylcholinesterase within the peripheral anionic subsite.

Cyclohexanoids namely, speciosin U, speciosin V and speciosin W from the endophytic fungus *Saccharicola* sp. showed acetylcholinesterase inhibitory activities comparable to reference inhibitor galantamine [12]. The IC_50_ value obtained for the most active compound speciosin U were 0.037 mg/mL and 0.026 mg/mL against acetylcholinesterase from human erythrocytes and from Electrophorus electricus, respectively. In the same experiments, the IC_50_ values obtained for galantamine were 0.076 and 0.0047 mg/mL against acetylcholinesterase from human erythrocytes and from *Electrophorus electricus*, respectively. The obtained results indicated that Speciosin U possessed higher in vitro inhibitory activity, comparable to the reference inhibitor galantamine against acetylcholinesterase from human erythrocytes. Therefore, the compound may be a good candidate for further in vitro and in vivo investigations.

The inhibition of α-amylase activity by *Salvia eriophora* leaf extracts containing active compounds salvigenin, fumaric acid and quercetagetin-3.6-dimethylether was evaluated [71]. Investigations were performed using p-nitrophenyl-D-glucopyranoside as the substrate. The sample was dissolved in a mixture of ethanol and water and mixed with an enzyme solution in phosphate buffer at pH 7.4. Next, for the initiation of the reaction, p-nitrophenyl-D-glucopyranoside was added, and the sample was incubated at 35 °C for 12 min. Then, p-nitrophenyl-D-glucopyranoside in phosphate buffer was added, and incubation was repeated at 37 °C. Absorbances were measured at 405 nm. The IC_50_ values determined by the procedure were 1.41 μg/mL for water extract and 8.88 μg/mL for methanolic extract [71]. Methanolic and aqueous extracts from the leaves of Salvia eriophora also inhibited both cholinesterases. The IC_50_ values obtained for methanolic and aqueous extracts against acetylcholinesterase were 9.91 and 15.06 μg/mL, respectively. Butyrylcholinesterase was inhibited by methanolic and aqueous extracts from the plant with IC_50_ = 5.17 and 10.82 μg/mL, respectively. The IC_50_ values obtained for tacrine used as positive standard against acetylcholinesterase and butyrylcholinesterase were 0.124 and 0.101 μg/mL, respectively. The antioxidant activity of Salvia eriophora leaf extracts was determined by DPPH, ABTS and N,N-Dimethyl-p-phenylenediamine (DMPD), using reduction of ferric and cupric ions assays. Both extracts showed similar antioxidant properties in all assays. For example, the IC_50_ values obtained in the DPPH assay were between 9 and 10 μg/mL; in the ABTS assay, they were about 6 μg/mL. The obtained IC_50_ values for the investigated extracts were also similar to those obtained for the most active positive control trolox. 

Iridoids, a large and still expanding class of cyclopentane pyran monoterpenes, are composed of two basic carbon frameworks, substituted iridoids and secoiridoids. They are more prevalent in plants and are rarely tested as acetylcholinesterase inhibitors. For investigations of acetylcholinesterase inhibition by extracts from arial parts of *Anarrhinum pubescens* containing iridoids belonging to the class of antirrhinosides, the HPTLC method was applied [20]. The detection of acetylcholinesterase inhibition was performed according to the Marston et al. method [20]. In the procedure, cetylcholinesterase was dissolved in Tris–hydrochloric acid buffer at pH 7.8; bovine serum albumin was added to the solution in order to stabilize the enzyme during the bioassay. TLC plates were eluted with an appropriate solvent (acetone or isopropanol) in order to wash them and were thoroughly dried immediately before use. After the migration of the sample in a suitable solvent, the TLC plate was dried and sprayed with enzyme stock solution and thoroughly dried again. For the incubation of the enzyme, the plate was incubated at 37 °C for 20 min. For the detection of the enzyme, solutions of 1-naphthyl in ethanol and of Fast Blue B salt in water were prepared immediately before use. After the incubation of the TLC plate, the naphthyl acetate solution and the Fast Blue B salt solution were mixed and sprayed onto the plate to give a purple coloration after 1–2 min. The high-resolution tandem mass spectrometry (HRMS/MS) analysis of the active acetylcholinesterase inhibitor was directly applied on the plate via HPTLC-MS coupling. Prior to sample development, the plate was washed twice with methanol–water, followed by heating at 100 °C on the TLC plate heater for 20 min. After the plate development, the compound was marked at UV 254 nm and automatically eluted via an elution-head-based interface. The eluted sample was directly transferred to a mass spectrometer [20]. The inhibition acetylcholinesterase activity of three compounds isolated from *Anarrhinum pubescens* was similar to the inhibition activity of rivastigmine.

Acetylcholinesterase inhibition potential by extracts obtained from leaves of *Maytenus distichophylla* and *Salacia crassifolia* contained triterpenes was evaluated by Ferreira et al. [29]. The in vitro anti-acetylcholinesterase activity of extracts and their constituents was determined using a 96-well microtiter plate following the Ellman’s method. For both investigated chloroform extracts, 3β,24-dihydroxyfriedelane, 3-oxo-28,29-dihydroxyfriedelane and 3 β-palmityloxy-urs-12-ene acetylcholinesterase inhibitory activities were observed [29]. The results showed that the crude extracts and some isolated terpenes have an acetylcholinesterase inhibition property similar to that presented by physostigmine and can be considered for further investigations.

Other examples of potential acetylcholinesterase activity inhibitors are presented in Table 1; butyrylcholinesterase activity inhibitors are in Table 2, and examples of antioxidant activity are presented in Table 3.

**Table 1:** Acetylcholinesterase inhibition by plant extracts.

Compounds	Plant Material	Acetylcholinesterase Inhibition Investigation Methods	Determination of Plant Extract Components Method	IC_50_	Reference
Gigantelline, gigantellinine and gigancrinine, cherylline- and crinine-type Amarylidaceae alkaloids	*Crinum jagus* bulbs	Ellman method with the application of 96-well microplate	Separation by preparative TLC and identification by NMR	From 1.83 μM for gigantellinine to 174.90 μM for sanguinine	[72]
Flavonol triglycosides	*Maytenus robusta* leaves	Ellman method with the application of 96-well microplate and docking study	TLC, LC-UV-Vis, LC-MS		[73]
Oxoprotoberberine alkaloid and flavone	*Miliusa thorelii* stem, root and leaves	Ellman method with the application of 96-well microplate and docking study	Separation by TLC and column chromatography and identification by NMR and MS	-	[74]
Diterpenoid alkaloids	*Aconitum anthoroideum* whole plant	Ellman method with the application of 96-well microplate	Separation by column chromatography and identification by NMR	From 0.065 μM for hetisinone to >100 μM for anthoroisine H	[75]
Monoterpene indole alkaloids	*Rauvolfia vomitoria* leaves	Ellman method with the application of 96-well microplate and docking study	Separation by TLC, column chromatography and identification by NMR	From 16.39 μM for rauvomitorine III to 186.62 μM for normacusine B	[76]
Polymethylated acylphloroglucinols	*Rhodomyrtus tomentosa* twigs and leaves	Ellman method with the application of 96-well microplate	Separation by semi-preparative HPLC and identification by NMR	From 8.68 μM for rhotomentosone E to 46.58 μM for rhodomentone A	[77]
Acylphloroglucinols	*Eucalyptus robusta* fruits	Ellman method with the application of 96-well microplate and docking study	Separation by semi-preparative HPLC and identification by NMR	From 2.55 μM for eucalyprobusones E and F to >40 μM for eucalyprobusal A–E and eucalyprobusone B	[78]
Flavanonol glucosides	*Agrimonia pilosa* Ledeb. aerial parts	Ellman method with the application of 96-well microplate	Separation by column chromatography and semi-preparative HPLC, identification by NMR	From 76.59 μM for (2S,3R)-dihydrokaempferol 3-O-b-D-glucoside to 97.53 μM for (2R,3S)-taxifolin 3-O-b- D-glucoside	[79]
Phenolic compounds	*Ficus sycomorus* L. leaf and stem bark	Ellman method with the application of 96-well microplate	LC/MS	-	[80]
Phenolic compounds, triterpenes	*Ocimum basilicum, Ocimum africanum, Ocimum americanum and Ocimum minimum* leaves	Ellman method with the application of 96-well microplate	UPLC–MS	From 2.571 mg/mL for *Ocimum americanum* to 31.42 mg/mL for *Ocimum minimum*	[81]
Alkaloids: galantamine, pseudolycorine, sanguinine and narciclasine	*Hippeastrum elegans* bulbs	Ellman method with the application of 96-well microplate	UPLC- MS and NMR	From 4.9 to 33.7 μg/mL for the different harvest time	[82]
Phenolic compounds	*Stachys annua* leaves	Ellman method	LC-MS/MS	119.8 μg/mL for methanolic and 150.1 μg/mL for water extracts	[83]
Phenolic compounds	*Rhaponticoides iconiensis* flowers, leaves and roots	Ellman method	LC-MS/MS and NMR	-	[84]
Phenolic compounds	*Sarcocephalus latifolius* bark	Ellman method with the application of 96-well microplate	GC-MS	-	[85]
Stilbenoids	*Rheum lhasaense* roots	Ellman method with the application of 96-well microplate	Semi-preparative HPLC, NMR	From 2.18 μM for 4′-methoxy-scirpusin A l to 1709 μM for resveratrol	[86]
(E)-β-caryophyllene, a-pinene, bicyclogermacrene, α-pinene, β-pinene and myrcene	*Gynura bicolor* leaves and stems	Ellman method	GC-MS	-	[87]
Coumarins, flavonoids and b-sitosterol	*Ferulago carduchorum* aerial parts	Ellman method with the application of 96-well microplate	TLC, column chromatography, NMR	For 39.64 μM for xanthotoxin to 854.05 μM for suberosin	[88]
Alkaloids	*Hippeastrum* *Vitattum* bulbs, *Hippeastrum striatum* bulbs, *Hippeastrum morelianum* bulbs, *Hippeastrum santacarina* bulbs, *Hippeastrum breviflorum* bulbs, *Hippeastrum glaucescens* bulbs and leaves, *Hippeastrum psittacinum* bulbs and leaves and *Rhodophiala* *bifida* bulbs	Ellman method with the application of 96-well microplate and molecular docking study	GC-MS, NMR	From 0.33 μg/mL for *Hippeastrum glaucescens* bulbs to 8.45 μg/mL for *Rhodophiala bifida* bulbs	[89]
Phenolic compounds	*Evolvulus alsinoides* leaves	Ellman method with the application of 96-well microplate	GC-MS	From 4.46 μg/mL for water extract to 7.55 μg/mL for chloroformic extract	[90]
Gallic acid derivative, hydroxybenzoic acid derivative and rutin	*Olax nana* whole plant	Ellman method	HPLC–DAD	33.2 μg/mL for crude methanolic extract	[91]
Phytol derivative and cinnamic acid ester	*Pycnanthus* *Angolensis* leaves	Ellman method with the application of 96-well microplate	TLC	22.26 μg/mL for eluptol and 6.51 μg/mL for omifoate A	[92]
Lycorine	*Hippeastrum goianum* seeds	Ellman method	HPLC–DAD	386.00 μg/mL for ethanol extracts obtained from dry leaves of plantlets produced by in vitro seed germination and 114.80 μg/mL for micropropagation of bulblets	[93]
Chlorogenic acid, limonene	*Crithmum maritimum* flowers, stems and leaves	Ellman method with the application of 96-well microplate	HPLC–DAD	-	[94]
Polyphenols	*Salvia pilifera* aerial parts	Ellman method with the application of 96-well microplate	LC-MS/MS	138.61 μg/mL for water extract and 94.93 μg/mL for methanol extracts	[95]
Phytol, neophytadiene, decamethylene dibromide, crodacid, stigma-5-en-3-ol	*Nonea micrantha* whole plant	Ellman method	GC-MS	From 44 μg/mL for n-hexane extract to 1035 μg/mL for chloroform extract	[96]
Phenylpropanoids, cinchonains, procyanidins	*Trichilia catigua* bark	Ellman method with the application of 96-well microplate	TLC, HPLC-DAD-MS/MS	From 142 μg/mL for hydroalcoholic extract to 346 μg/mL for hexane extract	[97]
Phenolic compounds	*Hypericum lydium* aerial part	Ellman method	GC-MS	-	[98]
Carvacrol, *p*-cymene, γ-terpinenecarvacrol, p-cymene and γ-terpinene	*Thymus algeriensis* leaves and *Teucrium polium* aerial parts	Ellman method with the application of 96-well microplate	GC-MS	98.84 μg/mL for *Thymus algeriensis* and 261.97 μg/mL for *Teucrium polium*	[99]
Chlorogenic acid, cynarin and arzanol	*Helichrysum* *Stoechas* flowers, stems, and leaves	Ellman method and molecular docking study	LC-MS/MS	260.7 μg/mL for flover extract and 654.8 7 μg/mL for stem/leaves extract	[100]
Phenolic compounds	*Zizyphus lotus* seeds	Ellman method	LC-MS/MS	0.88 mg/mL for phenolic yields of seeds extract	[101]
1,8-Cineole, l-Borneol and β-Pinène	*Rosmarinus* *Tournefortii* aerial parts	Ellman method	GC-MS	13.80 μg/mL for essential oil, 180.70 μg/mL for chloroform extract and >200 μg/mL for butanolic extract	[102]
Flavonoids, phenolic acids	*Cryptostephanus vansonii* roots, rhizomes, basal leaf region and leaves	Ellman method with the application of 96-well microplate	UHPLC–MS/MS	From 7.72 μg/mL for root extract to 25.58 μg/mL for leaf extract	[103]
Phenolic compounds	*Senecio biafrae* leaves	Ellman method	HPLC-DAD	347.22 μg/mL	[104]
Furanocoumarins	*Heracleum verticillatum, Heracleum sibiricum, Heracleum* *angustisectum,* and *Heracleum ternatum* leaves, fruits and roots	Ellman method with the application of 96-well microplate	NMR	From 0.30 μg/mL for *Heracleum verticillatum* roots extract to 42.4 μg/mL for *Heracleum ternatum* roots extract	[105]
γ-terpinene, carvacrol, p-cymene and β-caryophyllene	*Satureja thymbra* aerial parts and *Thymbra spicata* aerial parts	Ellman method	GC-MS	4.17 μg/mL for *Satureja thymbra* and 3.73 μg/mL for *Thymbra spicata*	[106]
Coumarins and flavonoids	*Citrus* *Aurantium* fructus	Ellman method with the application of 96-well microplate and molecular docking study	UHPLC–MS/MS	-	[107]
Salvigenin, fumaric acid, and quercetagetin-3.6-dimethylether	*Salvia eriophora* leaves	Ellman method	LC-MS/MS	15.06 μg/mL for water extract and 9.91 μg/mL for methanol extract	[108]
Phenolic compounds	*Ceratonia silique* leaves	Ellman method	HPLC-DAD	0.26 mg/mL	[109]
Phenolic compounds	*Muscari comosum* bulbs	Ellman method	HPLC-UV-Vis	107.64 μg/mL	[110]
Phenolic compounds	*Nymphaea pubescens* flowers	Ellman method	GC-MS	51.33 μg/mL for flower Extract, 380.77 μg/mL for pedicel extract	[111]
Alkaloids: epiberberine, skimmianine, palmatine, columbamine, jatrorrhizine	*Zanthoxylum nitidum* whole plant	Ellman method	HPLC-UV-MS	62.34 μg/mL for crude extract, 1745.34 μg/mL for ethanol extract and from 3.12 μg/mL for epiberberine to 34.82 μg/mL for jatrorrhizine	[71]
Phenolic compounds	*Solanum* *macrocarpon, Amaranthus viridis* and *Telfairia occidentalis* leaves	Ellman method with the application of 96-well microplate	HPLC	-	[112]
Flavonoids	*Eupatorium* *Lindleyanum*	Ellman method with the application of 96-well microplate	Spectrophotometric method	0.58 mg/mL	[113]
Phenolic compounds	*Mucuna pruriens* seeds	Ellman method	HPLC-DAD	0.27 mg/mL	[114]
α-pinene, β-pinene, limonene, α-caryophyllene, β-caryophyllene, caryophyllene oxide, α-bisabolol, myrcene and cannabidiol	*Cannabis sativa* flowering tops	Ellman method with the application of 96-well microplate	GC-MS	57.31 μg/mL for Chinese accession and 74.64 μg/mL for fibrante variety	[115]
Polymethylated phloroglucinol meroterpenoids	*Rhodomyrtus* *Tomentosa* twigs and leaves	Ellman method with the application of 96-well microplate	Column chromatography, NMR	22.9 μM for most active compound rhotomentodione D	[116]
Flavonoids	*Musa acuminate* leaves and fruits	Ellman method with the application of 96-well microplate	Column chromatography, HPTLC-EDA-Vis, NMR	404.4 μg/mL for ethyl acetate leaf extract to 1848.7 μg/mL for n-hexane fruit extract	[117]
Phenolic compounds	*Mentha pulegium* whole plant	Ellman method with the application of 96-well microplate	HPLC-DAD	1581 μg/L for aqueous extract	[118]
Catechins	*Camellia sinensis* leaves	Ellman method with the application of 96-well microplate	HPLC-DAD, NMR	42.38 μM for (−)-6-(5″R)-2″-ethoxy-3″,4″-dihydro-2H-pyrrole-epicatechin-3-gallate, 19.5 μM for epicatechin-3-gallate and 78.79 μM for epigallocatechin-3-gallate	[119]
Flavoalkaloids	*Camellia sinensis* leaves	Ellman method with the application of 96-well microplate	LC-DAD-MS	From 10.81 μM to 34.82 μM for most active compounds	[63]

**Table 2:** Butyrylcholinesterase inhibition by plant extracts.

Compounds	Plant	Butyrylcholinesterase Inhibition Investigation Methods	Determination of Plant Extract Components Method	IC_50_	Reference
Phenolic compounds	*Rhaponticoides iconiensis* flowers, leaves and roots	Ellman method	LC-MS/MS and NMR	-	[84]
Gallic acid derivative, hydroxybenzoic acid derivative and rutin	*Olax nana* leaves	Ellman method	HPLC–DAD	55.36 μg/mL for crude methanolic extract	[91]
Phytol derivative and cinnamic acid ester	*Pycnanthus* *Angolensis* leaves	Ellman method with the application of 96-well microplate	TLC	34.61 μg/mL for eluptol and 9.07 μg/mL for omifoate A	[92]
Polyphenols	*Salvia pilifera* aerial parts	Ellman method with the application of 96-well microplate	LC-MS/MS	99.13 μg/mL for water extract and 69.05 μg/mL for methanol extracts	[95]
Phytol, neophytadiene, decamethylene dibromide, crodacid, stigma-5-en-3-ol	*Nonea micrantha* whole plant	Ellman method	GC-MS	From 44 μg/mL for methanolic extract to 750 μg/mL for chloroform extract	[96]
Phenolic compounds	*Hypericum lydium* aerial part	Ellman method	GC-MS	-	[98]
Carvacrol, *p*-cymene, γ-terpinenecarvacrol, p-cymene and γ-terpinene, germacrene D, bicyclogermacrene), β-pinene and spathulenol	*Thymus algeriensis* leaves and *Teucrium polium* aerial parts	Ellman method with the application of 96-well microplate	GC-MS	124.09 μg/mL for *Thymus algeriensis* and 89.71 μg/mL for *Teucrium polium*	[99]
1,8-Cineole, l-Borneol and β-Pinène	*Rosmarinus* *Tournefortii* aerial parts	Ellman method	GC-MS	148.67 μg/mL for essential oil, 10.03 μg/mL for chloroform extract and 73.94 μg/mL for butanolic extract	[102]
Phenolic compounds	*Senecio biafrae* leaves	Ellman method	HPLC-DAD	378.79 μg/mL	[104]
γ-terpinene, carvacrol, p-cymene and β-caryophyllene	*Satureja thymbra* aerial parts and *Thymbra spicata* aerial parts	Ellman method	GC-MS	3.20 μg/mL for *Satureja thymbra* and 2.67 μg/mL for *Thymbra spicata*	[106]
Salvigenin, fumaric acid and quercetagetin-3.6-dimethylether	*Salvia eriophora* leaves	Ellman method	LC-MS/MS	10.82 μg/mL for water extract and 95.17 μg/mL for methanol extract	[108]
Phenolic compounds	*Mucuna pruriens* seeds	Ellman method	HPLC-DAD	0.18 mg/mL	[114]

**Table 3:** Antioxidant activity of plant extracts.

Compounds	Plant	Antioxidant Activity Investigation Methods	Determination of Plant Extract Components Method	IC_50_	Reference
Phenolic compounds	*Rhaponticoides iconiensis* lowers, leaves and roots	ABTS radical scavenging assay, DPPH radical scavenging assay, ferric reducing antioxidant power assay and cupric ion reducing/antioxidant power assay and metal chelating activity	LC-MS/MS and NMR	-	[84]
Phenolic compounds	*Sarcocephalus latifolius* bark	DPPH radical scavenging assay	GC-MS	From 0.098 mg/mL for hexane extract to 0.148 mg/mL for ethyl acetate extract	[85]
Phenolic compounds	*Evolvulus alsinoides* roots	DPPH radical scavenging assay and ferric reducing antioxidant power assay	GC-MS	Obtained by DPPH assay from 52.43 μg/mL for water extract to 117.45 μg/mL for petroleum ether extract; obtained by FRAP assay from 41.58 μg/mL for water extract to 115.72 μg/mL for petroleum ether extract	[86]
Gallic acid derivative, hydroxybenzoic acid derivative and rutin	*Olax nana* leaves	DPPH radical scavenging assay, ABTS radical scavenging assay, hydrogen peroxide free radical scavenging assays	HPLC–DAD	71.46 μg/mL by DPPH assay, 72.55 μg/mL by ABTS assay and 92.33 μg/mL by free radical assay	[91]
Chlorogenic acid, limonene	*Crithmum maritimum* aerial parts	DPPH radical scavenging assay, peroxide free radical scavenging assays and the ability of samples to stop the oscillations in Briggs– Rauscher assay	HPLC–DAD	-	[94]
Polyphenols	*Salvia pilifera* aerial parts	DPPH radical scavenging assay, ABTS radical scavenging assay and N,N-dimethyl-p-phenylenediamine dihydrochloride radical (DMPD^+^) scavenging assay	LC-MS/MS	8.56 μg/mL for water extract and 7.05 μg/mL for methanol extracts obtained by DPPH assay; 4.76 μg/mL for water extract and 3.52 μg/mL for methanol extracts obtained by ABTS assay; 30.95 μg/mL for water extract and 28.92 μg/mL for methanol extracts obtained by DMPD^+^	[95]
Phytol, neophytadiene, decamethylene dibromide, crodacid, stigma-5-en-3-ol	*Nonea micrantha*	DPPH radical scavenging assay, ABTS radical scavenging assay, total reducing power assay	GC-MS	3, 5, 93 and 120 μg/mL for aqueous, chloroform, n-hexane and methanolic extracts, respectively, obtained by DPPH assay; 60, 95, 100 and 150 μg/mL for ethyl acetate, aqueous, crude saponins and methanolic extracts, respectively obtained by ABTS assay	[96]
Phenylpropanoid, cinchonains, procyanidins	*Trichilia catigua* bark	DPPH radical scavenging assay	TLC, HPLC-DAD-MS/MS	From 43 μg/mL for hydroalcoholic extract to 60 μg/mL for chloroform extract	[97]
Phenolic compounds	*Hypericum lydium* aerial part	DPPH radical scavenging assay, ABTS radical scavenging assay	GC-MS	76.24 μg/mL for methanol extract and 168.64 μg/mL for water extract obtained by DPPH assay; 16.63 μg/mL for methanol extract and 20.48 μg/mL for water extract obtained by ABTS assay	[98]
Carvacrol, *p*-cymene, γ-terpinenecarvacrol, p-cymene and γ-terpinene	*Thymus algeriensis* leaves and *Teucrium polium* aerial parts	DPPH radical scavenging assay, ABTS radical scavenging assay, β-Carotene-linoleic acid bleaching assay, cupric ion reducing antioxidant capacity assay and total reducing power assay	GC-MS	For *Thymus algeriensis* 7.44 μg/mL by ABTS, 30.67 μg/mL by DPPH, 22.96 μg/mL by superoxide, 58.82 μg/mL reductive potential assay, 19.40 μg/mL by cupric ion reducing antioxidant capacity, 43.34 μg/mL by β-Carotene assay and for *Teucrium polium* 16.36 μg/mL by ABTS, 47.45 μg/mL by DPPH, 22.3 μg/mL by superoxide, 76.35 μg/mL reductive potential assay, 13.59 μg/mL by cupric ion reducing antioxidant capacity, 44.04 μg/mL by β-Carotene assay	[99]
Phenolic compounds	*Zizyphus lotus* seeds	DPPH radical scavenging assay and ferric reducing antioxidant power assay	LC-MS/MS	0.000067 mg/mL by DPPH assay, 2039.60 mg GAE/100 g by ferric reducing antioxidant power assay	[101]
1,8-Cineole, l-Borneol and β-Pinène	*Rosmarinus* *Tournefortii* aerial parts	DPPH radical scavenging assay, ABTS radical scavenging assay, superoxide radical scavenging assay by alkaline DMSO, reducing power assay, β-carotene/linoleic Acid bleaching assay, cupric reducing antioxidant capacity and ferrous ions chelating assay	GC-MS	129.28 to μg/mL by ferrous ions chelating assay and 314.13 μg/mL by β-carotene assay to >200 by other methods for essential oil, from 9.67 μg/mL by β-carotene assay to >200 μg/mL by ferrous ions chelating assay for chloroform extract and from 7.99 μg/mL by β-carotene assay to >200 μg/mL by ferrous ions chelating assay for butanolic extract	[102]
Phenolic compounds	*Senecio biafrae* leaves	DPPH radical scavenging assay, ABTS radical scavenging assay, hydroxyl radical scavenging assay, ferric reducing antioxidant power assay, NO radical scavenging assay and ferrous ions chelating assay	HPLC-DAD	78.25 μg/mL by ABTS, 92.08 μg/mL by DPPH, 22.3 μg/mL by hydroxyl radical scavenging assay, 127.23 μg/mL by NO radical scavenging assay, 118.76 μg/mL by ferrous ions chelating assay	[104]
Furanocoumarins	*Heracleum verticillatum, Heracleum sibiricum, Heracleum* *angustisectum, and Heracleum ternatum* leaves, fruits and roots	DPPH radical scavenging assay, ABTS radical scavenging assay	NMR	From 0.58 μg/mL for *Heracleum* *angustisectum* leaves extract to 45.76 μg/mL for *Heracleum sibiricum* fruits extract by DPPH assay; from 0.05 μg/mL for *Heracleum ternatum* fruits extract to 1.83 μg/mL for *Heracleum* *angustisectum* fruits extract by ABTS assay	[105]
γ-terpinene, carvacrol, p-cymene and β-caryophyllene	*Satureja thymbra* aerial parts and *Thymbra spicata* aerial parts	DPPH radical scavenging assay, ABTS radical scavenging assay, hydroxyl radical scavenging assay, ferric reducing antioxidant power assay, NO radical scavenging assay and metal chelating assay, cupric ion reducing assay	GC-MS	169.68 μg/mL for *Satureja thymbra* and 276.08 μg/mL for *Thymbra spicata* by ABTS, 475.53 μg/mL for *Satureja thymbra* and 121.94 μg/mL for *Thymbra spicata* by DPPH, 29.28 μg/mL for *Satureja thymbra* and 5.00 μg/mL for *Thymbra spicata* by hydroxyl radical scavenging assay, 3.22 μg/mL for *Satureja thymbra* and 4.89 μg/mL for *Thymbra spicata* by NO radical scavenging assay, 73.96 μg/mL for *Satureja thymbra* and 77.64 μg/mL for *Thymbra spicata* by ferric reducing assay, 127.27 μg/mL for *Satureja thymbra* and 138.38 μg/mL for *Thymbra spicata* by cupric ion reducing assay, 12.06 μg/mL for *Satureja thymbra* and 0.62 μg/mL for *Thymbra spicata* by metal chelating assay	[106]
Salvigenin, fumaric acid, and quercetagetin-3.6-dimethylether	*Salvia eriophora* leaves	DPPH radical scavenging assay, ABTS radical scavenging assay, N,N-Dimethyl-p-phenylenediamine radical scavenging assay (DMPD)	LC-MS/MS	9.94 μg/mL for water extract and 9.21 μg/mL for methanol extract by DPPH assay; 6.58 μg/mL for water extract and 6.03 μg/mL for methanol extract by ABTS assay; 38.10 μg/mL for water extract and 36.82 μg/mL for methanol extract by DMPD assay	[108]
Phenolic compounds	*Muscari comosum* bulbs	DPPH radical scavenging assay, NO radical scavenging assay and superoxide radical scavenging assay	HPLC-UV-Vis	36.73 μg/mL by DPPH assay, 144.13 μg/mL by NO radical scavenging assay and 54.15 μg/mL by superoxide radical scavenging assay	[110]
Flavonoids	*Eupatorium* *lindleyanum*	DPPH radical scavenging assay, superoxide radical scavenging assay, reducing antioxidant power assay and ferric reducing antioxidant power assay	Spectrophotometric method	37.13 μg/mL by DPPH assay, 19.62 μg/mL by superoxide radical scavenging assay, 81.22 μg/mL, 24.72 μg/mL by ferric reducing antioxidant power assay	[113]
Phenolic compounds	*Mucuna pruriens* seeds	DPPH radical scavenging assay, hydroxyl radical scavenging assay, free radical scavenging assays, ferrous ions chelating assay	HPLC-DAD	0.88 mg/mL by DPPH, 0.23 mg/mL by hydroxyl radical scavenging assay, 0.24 mg/mL by ferrous ions chelating assay	[114]
α-pinene, β-pinene, limonene, α-caryophyllene, β-caryophyllene, caryophyllene oxide, α-bisabolol, myrcene and cannabidiol	*Cannabis sativa* flowering tops	DPPH radical scavenging assay, free radical scavenging assays, ABTS radical scavenging assay, ferric reducing antioxidant power assay, β-carotene/linoleic acid bleaching assay, oxygen radical absorbance capacity assay	GC-MS	495.49 μg/mL for Chinese accession and 453.9 μg/mL for fibrante variety by DPPH assay; 840.4 μg/mL for Chinese accession and 155.98 μg/mL for fibrante variety by free radical scavenging assays; 24.354 μg/mL for Chinese accession and 16.09 μg/mL for fibrante variety by ABTS radical scavenging assay; 194.06 μg/mL for Chinese accession and 256.82 μg/mL for fibrante variety by β-carotene/linoleic acid bleaching assay; 1908.67 μg/mL for Chinese accession and 629.67 μg/mL for fibrante variety by ferric reducing antioxidant power assay; 3.39 μg/mL for Chinese accession and 2.11 μg/mL for oxygen radical absorbance capacity assay	[115]
Flavonoids	*Musa acuminate* fruits and leaves	DPPH radical scavenging assay, free radical scavenging assays, ABTS radical scavenging assay, hydroxyl radical scavenging assay	column chromatography HPTLC-EDA-Vis, NMR	From 9.0 μg/mL for ethyl acetate leaf extract to 589.8 μg/mL for n-hexane fruit extract by DPPH assay; from 187.3 μg/mL for ethyl acetate leaf extract to 1332.0 μg/mL for n-hexane fruit extract by ABTS assay; from 187.3 μg/mL for ethyl acetate leaf extract to 1332.0 μg/mL for aqueous methanol leaf extract by ABTS assay; from 10.2 μg/mL for crude extract leaf extract to 249.2 μg/mL for aqueous n-butanol fruit extract by hydroxyl radical scavenging assay	[117]
Phenolic compounds	*Mentha pulegium* whole plant	DPPH radical scavenging assay, ferric reducing antioxidant power assay, inhibition of lipids and protein oxidation assays	HPLC-DAD	35.71 μg/mL for aqueous extract, 38.09 μg/mL for methanolic extract by DPPH assay	[118]

## 3. Conclusions

With the increasing burden of Alzheimer’s disease, it is essential to discover and develop new treatment options capable of preventing and treating the disease. In recent years, many studies have been described on the search for substances of plant origin with potential activity against neurodegenerative diseases. For the complex nature of Alzheimer’s disease, multi-target-directed ligands are increasingly being considered as promising therapeutic agents for treatment of the disease. Recently, multitarget therapeutic strategies have been devised to target acetylcholinesterase, butyrylcholinesterase and, for example, monoamine oxidase B. Because, in Alzheimer’s disease brain, the acetylcholinesterase activity is maintained or repressed, while the butyrylcholinesterase activity tends to increase, the discovery of drugs inhibiting both enzymes as well as that of selective butyrylcholinesterase inhibitors is advisable. Many plant extracts also exhibit dual cholinesterase inhibitor activity and antioxidant properties, which is a promising prospect for the treatment of neurodegenerative diseases. 

This review was intended to provide a description of various medicinal plants, their extracts, fractions and active compounds that can be candidates for the treatment of Alzheimer’s disease. The available literature on various plant extracts and their active components has revealed that some have the potential to prevent or treat numerous diseases including neurodegenerative disorders. It is evident from the literature studies mentioned that the various plants and their components possess high in vitro activity against Alzheimer’s disease. Plant-derived compounds including alkaloids, flavonoids, saponins, phenolic acids and some other compounds can play a key role in the inhibition of neurodegenerative disorders.

Several Alzheimer’s disease targets are currently and intensively being investigated, divided in different hypotheses: mainly, cholinergic and β amyloid.

Most often, for in vitro investigations of acetylcholinesterase and butyrylcholinesterase activity inhibition, the spectrophotometric method firstly described by Ellman, currently most performed in 96-well microplates, is applied. Molecular docking is also a widely used technique in the search for new drugs, which reduces both the time and costs of lead investigations.

Further work is required to discover novel anti-Alzheimer’s disease active compounds and scale up the production of various biomolecules derived from these plants.

## 4. Future Directions

The in vitro anti-Alzheimer’s disease activity of many plant extracts and their active components suggests further detailed studies on these compounds to provide a platform for clinical trials that might lead to candidates of clinical applications. In many cases, these in vitro experiments ought to be extended to toxicological investigations and the determination of the bioavailability of the extracts and their active compounds.

Several medicinal plants listed in this review have demonstrated encouraging potential for Alzheimer’s disease treatment in in vitro experiments. This may lead to the development of novel therapeutic agents, which will provide alternative and complementary remedies that can be effective in preventing and treating the disease.

Alzheimer’s disease is caused by many factors. For this reason, the application of multitargeting compounds, able to concurrently tackle several pathogenetic pathways, is a promising tool in the treatment of the disease. 

Plant-derived compounds can be also exploited further by chemical derivatization and analog synthesis for a better pharmacokinetic approach and enhanced efficacies.

Further studies are required to decipher the protein targets and active compound–protein interactions in Alzheimer’s disease to successfully translate the experimental results into clinical effects. 

One of the limitations of transitioning the lead compounds into preclinical and clinical studies is to produce the desired compounds on a larger scale. Investigations on increasing the production of these compounds by plants as well as more effective methods of their extraction from plant materials are advisable. 

In many cases, although pre-clinical investigations have identified promising drug candidates for Alzheimer’s disease, clinical evidence is still pending.

## Abbreviations

NFκB	nuclear factor-κB,
APP	amyloid precursor protein
QSAR	quantitative structure–activity relationship
TLC	thin-layer chromatography
HPLC	high-performance liquid chromatography
UFLC–MS	ultrafiltration liquid chromatography–mass spectrometry
HPTLC-MS	high-performance thin-layer chromatography coupled with mass spectrometry
HPLC-DAD	high-performance liquid chromatography coupled with diode array detector
NMR	nuclear magnetic resonance
HRMS	high-resolution mass spectrometry
ESI	electrospray ionization
FLD	fluorescence detector
DPPH	2,2-diphenyl-1-picrylhydrazyl
ABTS	2,2’-azino-bis(3-ethylbenzothiazoline-6-sulfonic acid)
